# Development of Heterobimetallic Al/Mg Complexes for the Very Rapid Ring-Opening Polymerization of Lactides

**DOI:** 10.1021/acs.inorgchem.3c02410

**Published:** 2023-09-07

**Authors:** Marta Navarro, David González-Lizana, Luis F. Sánchez-Barba, Andrés Garcés, Israel Fernández, Agustín Lara-Sánchez, Ana M. Rodríguez

**Affiliations:** † Departamento de Biología y Geología, Física y Química Inorgánica, Universidad Rey Juan Carlos, 28933 Móstoles, Madrid, Spain; ‡ Departamento de Química Inorgánica, Orgánica y Bioquímica, Centro de Innovación en Química Avanzada (ORFEO-CINQA), Campus Universitario, Universidad de Castilla—La Mancha, 13071 Ciudad Real, Spain; § Departamento de Química Orgánica I and Centro de Innovación en Química Avanzada (ORFEO-CINQA), Facultad de Ciencias Químicas, Universidad Complutense de Madrid, 28040 Madrid, Spain

## Abstract

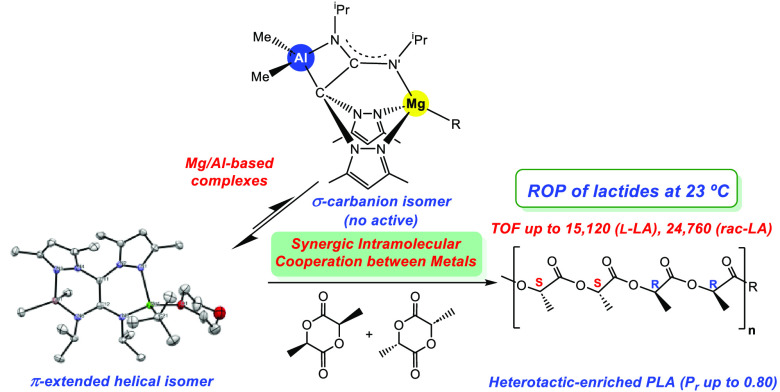

The successful architecture of active catalytic species with enhanced efficiencies is critical for the optimal exploitation of sustainable resources in industrially demanded processes. In this work, we describe the preparation of novel helical heterobimetallic Al/Mg-based complexes of the type [AlMe_2_(pbpamd^–^)MgR{κ^1^-O-(OC_4_H_8_O)}] [R = Et (**1a**), ^t^Bu (**2a**)] as potential catalysts. The design was performed through the sequential addition of the Al fragment to the ligand, followed by the Mg platform, resulting in a planar π-C_2_N_2_(sp^2^)–Al/Mg bridging core between metals. The new heterobimetallic species have been unambiguously characterized by single-crystal X-ray analysis. NOESY, DOSY, and EXSY NMR studies as well as density functional theory calculations corroborate both a rearrangement in solution to scorpionate complexes containing an unprecedented apical carbanion with a direct σ-C(sp^3^)–Al covalent bond named [{Mg(R)(pbpamd^–^) Al(Me)_2_}] [R = Et (**1b**), ^t^Bu (**2b**)] and an interconversion equilibrium between both isomers. We verified their utility and high efficiency as catalysts in the well-controlled ring-opening polymerization of the biorenewable l- and *rac*-lactide (LA) at 23 °C, reaching a remarkable turnover frequency value close to 25000 h^–1^ for *rac*-LA at this temperature and exerting a significant level of heteroselectivity (*P*_r_ = 0.80). Very interestingly, the kinetics demonstrate apparent first-order with respect to the catalyst and LA, which supports a synergic intramolecular cooperation between centers with electronic modulation among them.

## Abstract 2

Novel heterobimetallic Al/Mg complexes have been successfully designed through the sequential addition of both metal fragments, resulting in a planar π-C_2_N_2_(sp^2^)−Al/Mg bridging structure. These species mediate very rapidly the ring-opening polymerization of lactides (LAs) at 23 °C, reaching a remarkable TOF value of 24770 h^−1^ for *rac*-LA and exerting a significant level of heteroselectivity (*P*_r_ = 0.80). A synergic intramolecular cooperatization between centers with electronic modulation among them is responsible for these outperformed activities.

The development of heterometallic complexes is currently attracting great attention^1^ because two different metals remain in the same molecular environment and can operate together to create a “beneficial” (cooperative) effect, resulting in a final synergic outcome “greater than the sum of their parts”. Thus, heterometallic cooperativity between active centers represents an emerging strategy to enhance the catalytic activity^2^ and selectivity in both small and macromolecular transformations.^3^

Commonly, heterometallic species can operate via multisite interactions, where each metal catalyzes different reaction steps^1^ or with one metal acting as the principal catalytic site and the other metal(s) modulating its reactivity.^4^

Thus, it has been extensively reported that heterodinuclear catalysts outperform their monometallic analogues or mixtures of them in many catalytic processes. For instance, certain combinations have displayed higher activities in the catalytic preparation of oxygenated molecules and polymers, including poly(lactide)s (PLAs),^5^ cyclic carbonates,^6^ and poly(carbonate)s.^7^ However, the mere preparation of heterodinuclear complexes with different metals is not sufficient to confer a synergic performance.^7a^

On the other hand, our research group has been interested over the past few years in the sustainable ring-opening polymerization (ROP) of cyclic esters^8^ considering their promising applications, including the controlled release of drugs,^9^ regenerative medicine,^10^ and wound healing,^11^ as well as in packaging and agriculture.^12^ In this context, a reduced number of complexes, including a combination of alkali metals (K/Li/Na) and divalent (Mg/Zn),^5c,13^ trivalent (Al/In/Y)^14,5b^ or tetravalent (Ge/Sn) elements,^15^ and, less commonly, divalent/trivalent, transition metal/main group, and f-block heterocombinations,^1^ have been reported for the ROP of lactides (LAs). Furthermore, the incorporation of earth-abundant, low-toxicity, and biologically benign metals in these catalysts, such as Mg and Al, offers an environmentally and economically attractive route to combine the high activity of Mg with the control offered by Al systems. Regrettably, the preparation of heterobimetallic Al/Mg-based catalysts remains scarcely explored due to the different physical and chemical properties of the metals. In fact, only the inspiring successful work recently reported by Garden et al.^5b^ describes the design of salen-type Al/Mg-based catalysts, and their cooperative performance in the ROP of *rac*-LA, improving their activity by a factor of up to 11 compared to their mono-Al analogues. Unfortunately, the employment of an initiator (propylene oxide) and harsh conditions (120 °C) were required to reach high monomer conversions in a few minutes, leading to atactic PLAs.

With this aim in mind, now we take the challenge of designing Al/Mg complexes that allow a synergic intramolecular cooperation between centers to display enhanced efficiencies in the well-behaved ROP of LAs under very mild conditions.

Initially, we designed a strategy consisting of the sequential addition of an excess of Grignard reagent (3 equiv) to our previously reported NH-tautomer [AlMe_2_(κ^2^-pbpamd)],^16^ which yielded the heterobimetallic Al/Mg complexes [AlMe_2_(pbpamd^–^)MgR{κ^1^-O-(OC_4_H_8_O)}] [R = Et (**1a**), ^t^Bu (**2a**)] as pale-yellow solids in good yields (ca. 70%; Scheme 1).

**Scheme 1:**
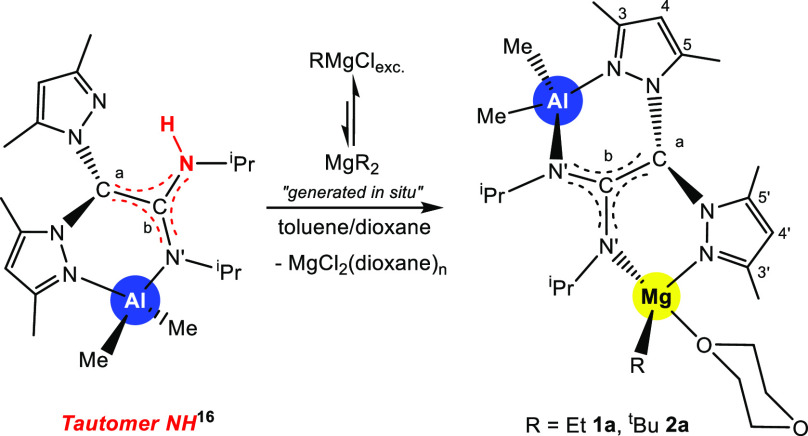
Synthesis of the Heterobimetallic Mg/Al Complexes **1a** and **2a**

The ^1^H and ^13^C{^1^H} NMR spectra of complexes **1a** and **2a** in benzene-*d*_6_ at room temperature (Figures S1 and S2) present two sets of resonances for the pyrazole rings and the amidinate substituents, indicating both a bidentate fashion of the amidinate fragment and asymmetry of the molecule (Scheme 1). In addition, the disappearance of the singlet for the N–H group in the ^1^H NMR spectra, in conjunction with maintenance of the C^a^ shift in the ^13^C {^1^H} NMR spectra (∼90 ppm) provide solid evidence for the presence of an extended π-C_2_N_2_(sp^2^)–Al/Mg core, as previously observed in our Al/Al^16^ and Mg/Mg^17^ homodinuclears. Moreover, one broad singlet appears for the terminal dioxane molecule and three sets of signals at higher field for the alkyl Mg–R and Al–Me_2_ groups.

Interestingly, the restricted rotation around the C^a^–C^b^ bond in complexes **1a** and **2a** leads to an inherent helical chirality,^18^ with the formation of a racemic mixture of the *M* and *P* enantiomers, as previously observed in the related homobimetallics.^16,17^ Also, the formation of a new sterogenic center at the Mg atom occurs in **1a** and **2a**, affording two diastereoisomers (Figure S3). These complexes show a rapid dynamic exchange at room temperature, which can further reversibly coordinate from both sides of the planar arrangement in the Mg center (Scheme 1), as previously observed in our tetranuclear Mg apical complexes.^17^ Thus, the variable-temperature ^1^H NMR studies in toluene-*d*_8_ (from +50 to −20 °C) for **1a** show a coalescence temperature, *T*_c_, of 293.15 K and a free-energy value, Δ*G*^⧧^, of 61 kJ·mol^–1^, for the protons H^4,4′^, showing a double set of singlets corresponding to the two possible pairs of enantiomers (Figure S4).

More importantly, complexes **1a** and **2a** present a dynamic interconversion in a benzene-*d*_6_ solution at ambient temperature to another nonisolable isomer corresponding to an unprecedented apical carbanion κ^3^-scorpionate with a direct σ-C(sp^3^)–Al covalent bond of the type [MgR(pbpamd^–^)AlMe_2_] [R = Et **1b**), ^t^Bu (**2b**); see Figure S2 for complexes **2a** and **2b**], as a result of the lability of the dioxane molecule. A preliminary assessment of this equilibrium confirms the high dependence on the donor solvent concentration; i.e., an excess of tetrahydrofuran benefits the π-extended arrangement in **1a** and **2a** (Figure S5), possibly due to the higher Lewis basicity of the O-donor atom than the pyrazole N atom. The arrangements proposed for **1a**, **1b**, **2a**, and **2b** were additionally confirmed by ^1^H NOESY-1D NMR experiments (Figure S6).

In addition, the DOSY spectrum for complex **2a** shows two different sets of signals for two species with different diffusion order, indicating the existence in solution of isomers **2a** and **2b** (Figure S7). Conversely, the 2D EXSY experiment for this complex corroborates a dynamic exchange equilibrium between both isomers (Figure S8). Furthermore, density functional theory (DFT) calculations for complex **2** support the arrangement proposed for isomer **2b**, in view of the good agreement found when the experimental and computed ^1^H and ^13^C NMR shifts were compared (Figure S9 and Tables S1–S3).

X-ray diffraction studies for complex **2a** revealed a monomeric dinuclear structure (Figure 1; crystallographic details for **2a** are given in Table S4) and confirmed a centrosymmetric unit cell, with the Mg(1) atom being a stereogenic center and the inherent helical chirality in the ligand (pbpamd^–^), given the restricted rotation around the C(11)–C(12) bond. These studies also showed that the presence in solution of the equimolecular mixture for the two diastereoisomers in **2a** was maintained in the solid state (*MS* + *PR* and *MR* + *PS*; Figures S3 and S10). Both the Al and Mg centers present a distorted tetrahedral geometry and are bridged by one single ligand, which is in a κ^2^-N,N′;κ^2^-N,N′ coordination mode. More interestingly, the planar π-extended C_2_N_2_ system is evidenced by both the angles close to 120° around C(11) and C(12) atoms and the dihedral angles N(6)–C(12)–C(11)–N(4) and N(5)–C(12)–C(11)–N(2) (15.52° and 13.72°, respectively), as well as the C(11)–C(12) bond length, which is intermediate between a single and a double C–C bond (∼1.339–1.455 Å).

**Figure 1:**
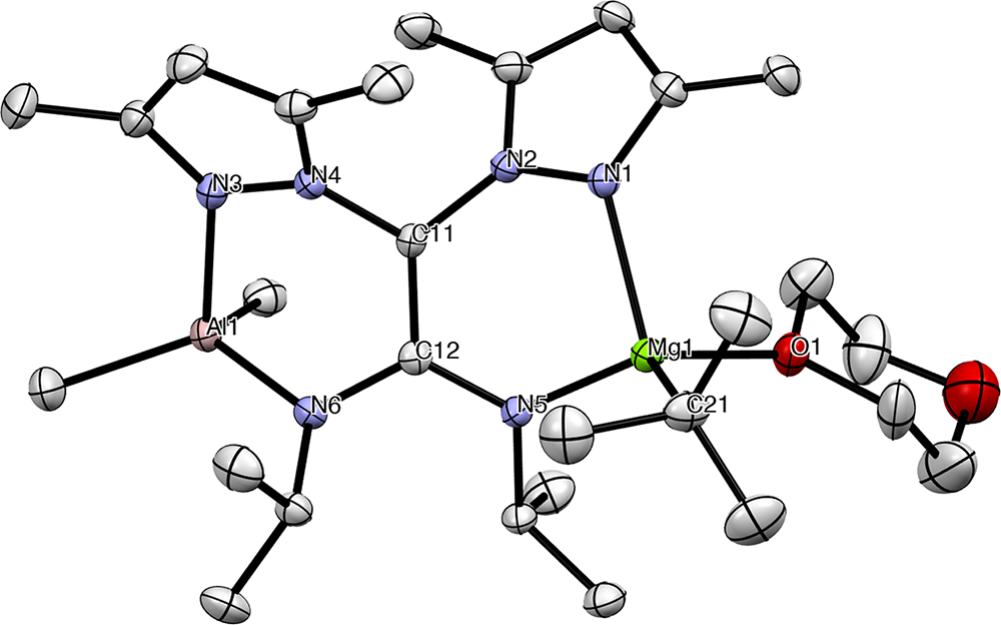
ORTEP view of the *M* diastereisomer of **2a** with 30% probability ellipsoids. Selected bond lengths (Å) and angles (deg): Mg(1)–N(1) = 2.122(1); Mg(1)–N(5) = 2.034(1); Al(1)–N(6) = 1.862(1); Al(1)–N(3) = 1.981(1); N(5)–C(12) = 1.355(2); N(6)–C(12) = 1.396(2); C(11)–C(12) = 1.390(2); C(12)–C(11)–N(4) = 122.8(1); C(12)–C(11)–N(2) = 124.2(1); N(4)–C(11)–N(2) = 113.0(1); N(5)–C(12)–N(6) = 123.6(1); C(11)–C(12)–N(5) = 120.1(1); C(11)–C(12)–N(6) = 116.2(1).

In a further stage, we focused our attention on the potential utility of **1a** and **2a** as catalysts for a sustainable and industrially demanded process such as the ROP of l- and *rac*-LA (Table 1) under different conditions.

**Table 1:**
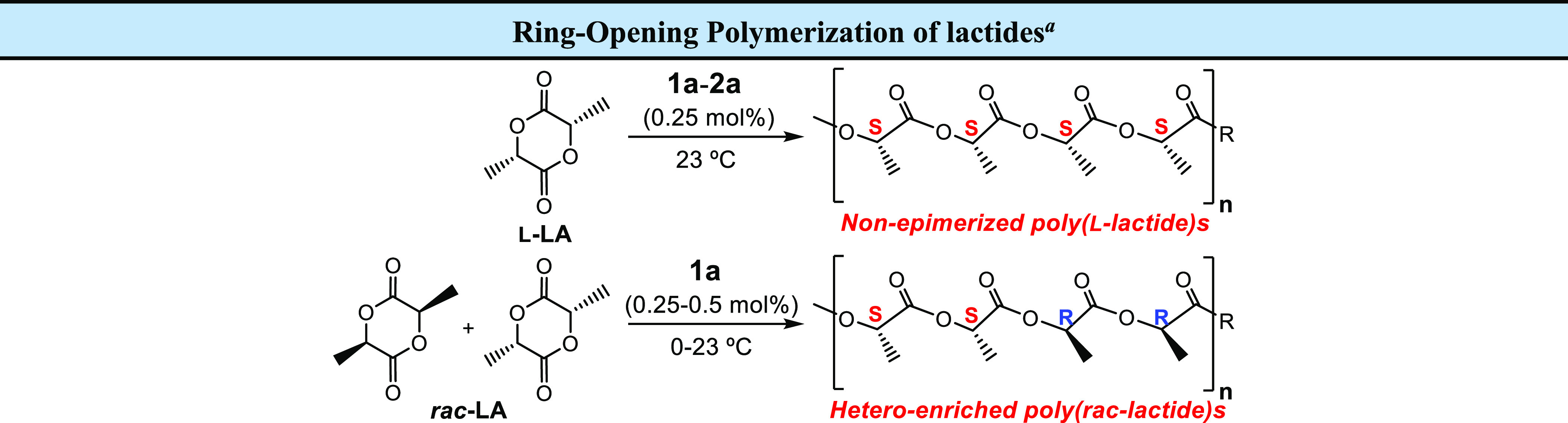
ROP of l- and *rac*-LAs Mediated by Catalysts **1a** and **2a**

entry	Cat.	monomer	Cat.:monomer	time (min)	conv. (%)^b^	TOF (h^–1^)^c^	*M*_n,theo_ (Da)^d^	*M*_n,GPC_ (Da)^e^	*Đ*_M_^e^	*P*_s_^f^
1	**1a**	l-LA^g^	1:400	1	63	15120	36300	3500	1.08	
2	**1a**	l-LA^g^	1:400	1.6	98	14700	56500	58100	1.10	
3	**2a**	l-LA^g^	1:400	2	24	2800	13800	14400	1.04	
4	**1a**	*rac*-LA	1:200	20 s	60	21600	17300	16100	1.09	0.74
5	**1a**	*rac*-LA	1:400	25 s	43	24770	24800	25900	1.10	0.74
6	**1a**	*rac*-LA	1:400	1	96	23040	55300	54500	1.12	
7	**1a**	*rac*-LA^h^	1:400	1	26	6240	15000	13800	1.04	0.80

a Polymerization conditions: 46 μmol of catalyst, 23 °C, [l- and *rac*-LA]_0_ = 1.0 M in tetrahydrofuran as the solvent, unless specified otherwise.

b The percentage conversion of the monomer was calculated by the ^1^H NMR polymer/unreacted monomer ratio.

c TOF (turnover frequency) = number of moles of starting material consumed/(moles of catalyst × time of reaction).

d Theoretical *M*_n_ = (monomer/initiator) × (% conversion) × (*M*_w_ of LA).

e Determined by GPC relative to polystyrene standards in tetrahydrofuran. Experimental *M*_n_ was calculated considering Mark–Houwink corrections^21^ for *M*_n_ [*M*_n_(obsd) = 0.58 × *M*_n_(GPC)].

f *P*_s_ is the probability of racemic linkages between monomer units.^22^

g [l-LA]_0_ = 0.4 M in toluene.

h Reaction temperature at 0 °C.

Thus, **1a** acted as a very active single-component initiator in toluene at room temperature, and 63% of 400 equiv of l-LA were transformed in only 1 min, with complete monomer conversion after 1.6 min, to produce medium-low molecular weight PLA materials with very narrow dispersities (Table 1, entries 1 and 2; *M*_n_ = 35000–58000; *Đ*_M_ = 1.08–1.10), reaching very high TOF values of 15100–14700 h^–1^. Catalyst **2a** exhibits a significantly reduced activity, possibly due to the higher steric hindrance of the ^t^Bu alkyl group, which might hamper the initiation step, reaching 24% conversion in 2 min to produce PLAs with similar dispersities (Table 1, entry 3; *M*_n_ = 14400; *Đ*_M_ = 1.04).

These outperformed activities encouraged us to inspect the ROP of *rac*-LA employing catalyst **1a** at room temperature in tetrahydrofuran, given the limited solubility of this mixture of stereoisomers in toluene at this temperature. We were delighted to find that **1a** also displayed very high activity and transformed 60% of 200 equiv of this monomer after only 20 s, showing a TOF value of 21600 h^–1^ (Table 1, entry 4; *M*_n_ = 16100; *Đ*_M_ = 1.09). Strikingly, this catalyst was also capable of converting 43% of 400 equiv of *rac*-LA after 25 s, reaching near-complete conversion in 1 min, under identical reaction conditions, with outperformed TOFs of 24760–23040 h^–1^. Moreover, catalyst **1a** showed moderate activity (26%) even at 0 °C, with very narrow dispersity (Table 1, entries 5–7).

To the best of our knowledge, these activity values, although lower than those recently reported for highly efficient heterobimetallic combinations in the ROP of *rac*-LA (i.e., K/Mg or K/Ca transforms 85 equiv of LA in 5 s at room temperature),^5a^ are the highest values reported to date for an Al-based heterobimetallic species in the ROP of *rac*-LA at room temperature.^5b^ It is also worth noting that this activity value found for **1a** outperforms those obtained in our group by Al- and Mg-based mononuclear systems ([Al(Me)_2_(κ^2^-pbpamd)],^19^ 75% conversion at 70 °C after 18 h; [Mg(Me)(κ^3^-pbpamd)],^20^ 42% conversion at 70 °C after 72 h) or mixtures of them, suggesting a synergic intramolecular cooperation between centers.

In addition, the experimental *M*_n_ values showed good agreement with those expected considering one molecule of catalyst per polymer chain (Table 1, entries 1–7), indicating that both centers do not act independently (see the kinetic studies below to confirm this hypothesis). Moreover, the very narrow dispersities observed indicate well-controlled living propagations and the existence of a single type of reaction site.

Furthermore, the inspection of low-molecular-weight PLAs by matrix-assisted laser desorption/ionization time-of-flight mass spectrometry (Figure S11) provided evidence that the ring opening of *rac*-LA occurs by the initial addition of an alkyl (Mg–R) fragment to the monomer.

Despite the low steric hindrance of this ligand, microstructural analysis of the PLAs revealed that **1a** exerts a significant preference for heterotactic dyad enchainment at room temperature, which is successfully increased at 0 °C (Table 1, entry 4, *P*_s_ = 0.74; entry 5, *P*_s_ = 0.80; Figure S12a,b), as a consequence of the rigid structure of the catalyst through the π-C_2_N_2_(sp^2^)–Al/Mg fragment.

Finally, kinetic studies conducted for the ROP of l-LA employing **1a** at room temperature in toluene unambiguously confirmed a pseudo-first-order dependence with respect to the monomer and catalyst concentrations (square correlation coefficients ≥ 0.97; Figures S13 and S14 and Table S3), evidencing that both metals do not act independently and the occurrence of a synergic intramolecular cooperation between centers.

In conclusion, we present herein the successful design and unambiguous characterization of novel heterobimetallic Al/Mg complexes having a π-C_2_N_2_(sp^2^)–Al/Mg bridging core, as potential catalysts in the ROP of LA.

NOESY, DOSY, and EXSY NMR studies as well as DFT calculations reveal both a rearrangement in solution into scorpionate complexes containing an unprecedented apical carbanion with a direct σ-C(sp^3^)–Al covalent bond and an interconversion equilibrium between both isomers.

Very importantly, we verified their utility and high efficiency for the well-controlled ROP of l- and *rac*-LA at room temperature (23 °C), reaching a remarkable TOF value close to 25000 h^–1^ for *rac*-LA and exerting a significant preference for heterotactic dyad enchainment (*P*_r_ = 0.80).

In addition, kinetic investigations established an apparent reaction first order with respect to the catalyst and monomer concentrations, which supports a synergic intramolecular cooperation between centers with electronic modulation among them, which justifies their outperformed activity in comparison with their Al/Mg mononuclear and even their homodinuclear counterparts.

Further work is ongoing in our laboratories not only to comprehend which catalyst features are decisive for the rational design of synergically active Al/M(II)-based catalysts but also to find new opportunities in additional demanded catalytic processes.

## Supporting Information Available

Preparation, spectroscopic, structural, dynamic solution, and DFT calculation details of complexes as well as an experimental description and kinetic investigations for the preparation of poly(l-LA) and poly(*rac*-LA) (PDF)

## Dedication

Dedicated to Dr. Juan Fernández on the occasion of his retirement.
