# Supplementary material for: Development of Heterobimetallic Al/Mg Complexes for the Very Rapid Ring-Opening Polymerization of Lactides
Source: Inorg Chem. 2023 Sep 7;62(37):14833–7. doi: 10.1021/acs.inorgchem.3c02410 (PMC10521010; doi:10.1021/acs.inorgchem.3c02410)
Supplement: Supplementary file 1 — ic3c02410_si_001.pdf [file ic3c02410_si_001.pdf]

SUPPORTING INFORMATION TO

Development of Heterobimetallic Al-Mg Complexes for the Very Rapid ROP of Lactides

Marta Navarro,[a,b] David González-Lizana,[a] Luis F. Sánchez-Barba,[,a] Andrés Garcés,[*,a] Israel Fernández,[c] Agustín Lara-Sánchez,[b] and Ana M. Rodríguez[b]*

[a]Departamento de Biología y Geología, Física y Química Inorgánica, Universidad Rey Juan Carlos, Móstoles-28933-Madrid, Spain.

E-mail: luisfernando.sanchezbarba@urjc.es; andres.garces@urjc.es

[b]Universidad de Castilla-La Mancha, Departamento de Química Inorgánica, Orgánica y Bioquímica- Centro de Innovación en Química Avanzada (ORFEO-CINQA), Campus Universitario, 13071-Ciudad Real, Spain.

[c]Departamento de Química Orgánica I and Centro de Innovación en Química Avanzada (ORFEO-CINQA), Facultad de Ciencias Químicas, Universidad Complutense de Madrid, 28040, Madrid, Spain.

SUPPORTING INFORMATION TO

Table of Contents

1. Materials and Methods

General Procedures S5

Instruments and Measurements S5

2. Preparation details of complexes 1a,b–2a,b

Synthesis of complexes **1a**,**b**–**2a**,**b** S7

3. Spectroscopic details

Figures S1–S2. ^{1}H and ^{13}C-^{1}H NMR spectra of complexes **1a**,**b**–**2a**,**b** S9

4. Structural studies

Figure S3. Possible pairs of enantiomers of **1a**,**b**–**2a**,**b** S11

5. Dynamic behavior studies

Figure S4. VT ^{1}H NMR spectra in toluene-d_8 of **1a** S12

6. Dependence on the donor solvent concentration

Figure S5. ^{1}H NMR spectra of complexes **1a** with an excess of tetrahydrofuran S13

7. 1D and 2D 1H NMR solution studies: selective 1D NOESY, DOSY and EXSY Experiments

Figure S6. 1D NOESY responses for complexes **2a** and **2b** S14

Figure S7. DOSY responses for complexes **2a** and **2b** S15

Figure S8. 2D EXSY responses for complexes **2a** and **2b** S16

8. DFT calculations for complex 2

Computational details S17

Thermodynamic stability study of the possible reorganization products of complex **2a** in toluene S17

Table S1. Gibbs free energies values calculated at 298.15 K of optimized structures at the PCM(toluene)-B3LYP-D3/def2-TZVP//PCM(toluene)-B3LYP–D3/def2-SVP level for the different ligand arrangements in heterodinuclear aluminum-magnesium complexes S18

Input coordinates S19

Expanded ^{13}C-{^{1}H}-NMR spectra of complex [$AlMe_2$(pbpamd^{-})Mg^{t}Bu{κ^1-O-(OC_4H_8O)}] **2** S30

Figure S9. Expanded area of ^{13}C{^{1}H}-NMR spectrum for complex **2** S30

Table S2. Experimental and Computed ^{13}C{^{1}H}-NMR chemical shifts for C^a and C^b atoms in complexes **2a**, **2b** and hypothetical **2b**-Mg S31

Table S3. Experimental and Computed ^{13}C{^{1}H}-NMR chemical shifts differences in ppm between **2a** and **2b** or hypothetical **2b**-Mg for C^a and C^b atoms S31

9. X-Ray Diffraction Studies: Crystallographic Structure Determination for Complex **2a**.

Details for crystallographic studies and structural refinement S32

Table S4. Crystal data and structure refinement for **2a** S33

Figure S10. ORTEP views of *P* and *M* enantiomers and their alternating disposition along the c axis in the unit cell of **2a** S34

10. Experimental details for the ring-opening polymerization of poly(lactide)s

General procedures for catalytic experiments S35

Figures S11. Selected areas of MALDI-ToF mass spectrum of poly(*rac*-lactide) synthesized by **1a** S36

Figures S12. Homodecoupled ^{1}H NMR spectra of poly(*rac*-lactide)s prepared by **1a** S37

11. Kinetic investigations for the ring-opening polymerization of poly(*rac*-lactide)s

Typical kinetic experiment procedure S38

Kinetics analysis S39

Figures S13. Pseudo-first-order kinetic plot for *rac*-LA polymerization employing **2a** S39

Figures S14. Plot of ln k_{app} versus ln [**2a**]$_0$ for *rac*-LA polymerization S40

Table S5. Rate constant dependence on the initial catalysts concentration for **2a** S41

SUPPORTING INFORMATION TO

12. References S42

SUPPORTING INFORMATION TO

1. Materials and methods

General procedures

All manipulations were carried out under a nitrogen atmosphere using standard Schlenk techniques or a glovebox. Solvents were pre-dried over sodium wire and distilled under nitrogen from sodium (toluene and *n*-hexane) or sodium-benzophenone (dioxane). Deuterated solvents were stored over activated 4 Å molecular sieves and degassed by several freeze-thaw cycles. The protioligand Hpbpamd was prepared according to the literature procedures.[1] The Grignard reagents RMgCl (R= Et, ^{t}Bu) and $AlMe_3$ were used as purchased (Aldrich). L-Lactide and *rac*-lactide were sublimed twice, recrystallized from THF, and finally sublimed again prior to use. All kinetics experiments were repeated at least twice and were mutually consistent.

Instruments and measurements

NMR spectra were recorded on a Bruker BioSpin GmbH (^{1}H NMR 500 MHz and ^{13}C NMR 125 MHz) spectrometer and were referenced to the residual deuterated solvent signal. Microanalyses were performed with a Perkin-Elmer 2400 CHN analyzer. ^{1}H NMR homodecoupled and NOESY-1D spectra were recorded on the same instrument with the following acquisition parameters: irradiation time 2 s and 256 scans, using standard BRUKER-FT software. 2D NMR spectra were acquired using the same software. Gel permeation chromatography (GPC) measurements were performed on a Polymer Laboratories PL-GPC-220 instrument equipped with a PLgel 5 Å Mixes-C column, a refractive index detector, and a PD2040 light-scattering detector. The GPC column was eluted with THF at 40 °C at 1 mL/min and was calibrated using eight monodisperse polystyrene standards in the range 580-483 000 Da. MALDI-ToF MS data were acquired with a Bruker Autoflex II ToF/ToF spectrometer, using a nitrogen laser source (337 nm, 3 ns) in linear mode with a positive acceleration voltage of 20 kV. Samples were prepared as follows: PLA (20 mg) was dissolved in HPLC quality THF with a matrix and NaI in a 100:5:5 ratio. Before evaporation, 10 μL of the mixture solution was deposited on the sample plate. External calibration was performed by using Peptide Calibration

Standard II (covered mass range: 700−3 200 Da) and Protein Calibration Standard I (covered mass range: 5 000−17 500 Da). The microstructures of PLA samples were determined by examination of the methine region in the homodecoupled ^{1}H NMR spectrum of the polymers recorded at room temperature in $CDCl_3$ on a Bruker BioSpin GmbH spectrometer with concentrations in the range 1.0 to 2.0 mg/mL.

SUPPORTING INFORMATION TO

2. Preparation details of complexes 1a,b–2a,b

Synthesis of complex [$AlMe_2$(pbpamd$^-$)MgEt{κ^1-O-(OC_4H_8O)}] (1)

In a 100 mL Schlenk tube, Hpbpamd (1.00 g, 3.03 mmol), was dissolved in dry toluene (30 mL) and cooled to -70 ºC. A solution of $AlMe_3$ (2.0 M in toluene) (1.51 mL, 3.03 mmol) was added and the mixture was allowed to warm up to 20 ºC and stirred during 1 h. Dry dioxane was added on a 1:2 ratio (dioxane/toluene) and the mixture was cooled to -30 ºC. A solution of EtMgCl (2.8 M in toluene) (3.24 mL, 9.09 mmol) was added and the mixture was allowed to warm up to 20 ºC and stirred for 18 h. The volatiles were removed under *vacuum*, the resulting yellow solid extracted with *n*-hexane (1×100 mL) and filtered. The obtained solution was cooled to -26 ºC to afford **1a** as a yellow semicrystaline solid. Yield: 72 %, 1.15 g. Anal.Calcd. (%) for $C_{26}H_{47}AlMgN_6O_2$: C, 59.26; H, 8.99; N, 15.95. Found: C, 59.40; H, 9.03; N, 15.99.

1a. **^{1}H-NMR** (500 MHz, C_6D_6, 297 K). δ 5.40 (s, 1 H, H^4), 5.37 (s, 1 H, $H^{4'}$), 4.04 [sept, $^3J_{H-H}$ = 6.2 Hz, 1 H, NC*<u>H</u>*(CH_3)$_2$], 3.71 [sept, $^3J_{H-H}$ = 6.2 Hz, 1 H, NC*<u>H</u>*(CH_3)$_2$], 3.39 (*br*, 8 H, OC_4H_8O), 2.19 (s, 3 H, Me^5), 2.08 (s, 3 H, $Me^{5'}$), 1.60 (s, 3 H, Me^3), 1.59 [d, $^3J_{H-H}$ = 6.2 Hz, 3 H, NCH(C*<u>H</u>*$_3$)$_2$], 1.50 [t, $^3J_{H-H}$ = 8.0 Hz, 3 H, $MgCH_2$C*<u>H</u>*$_3$], 1.50 [d, $^3J_{H-H}$ = 6.2 Hz, 3 H, NCH(C*<u>H</u>*$_3$)$_2$], 1.36 (s, 3 H, $Me^{3'}$), 1.21 [d, $^3J_{H-H}$ = 6.2 Hz, 3 H, NCH(C*<u>H</u>*$_3$)$_2$], 1.16 [d, $^3J_{H-H}$ = 6.2 Hz, 3 H, NCH(C*<u>H</u>*$_3$)$_2$], 0.18 [q, $^3J_{H-H}$ = 8.0 Hz, 2 H, MgC*<u>H</u>*$_2$$CH_3$], –0.04 [s, 3 H, Al(C*<u>H</u>*$_3$)$_2$], –0.14 [s, 3 H, Al(C*<u>H</u>*$_3$)$_2$]. **^{13}C-{^{1}H}-NMR** (125 MHz, C_6D_6, 297 K), δ 162.9 (C^b), 147.7, 145.4, 143.5, 140.7 ($C^{3,3' \text{ or } 5,5'}$), 105.6 ($C^4$), 105.3 ($C^{4'}$), 92.4 ($C^a$), 66.8 ($C_4H_8O_2$), 48.8 [N*<u>C</u>*H($CH_3$)$_2$], 47.4 [N*<u>C</u>*H($CH_3$)$_2$], 27.7, 26.8, 25.8, 25.6 [NCH(*<u>C</u>*H_3)$_2$], 25.2 ($MgCH_2$*<u>C</u>*H_3), 14.0 (Me^3), 13.9 ($Me^{3'}$), 10.6 (Me^5), 10.4 ($Me^{5'}$), –1.0 (Mg*<u>C</u>*H_2CH_3), –5.4, –8.0, [*br*, $AlMe_2$]. **1b**. **^{1}H-NMR** (500 MHz, C_6D_6, 297 K), δ 5.26 (s, 1 H, H^4), 3.90 [sept, $^3J_{H-H}$ = 6.3 Hz, 1 H, NC*<u>H</u>*(CH_3)$_2$], 3.76 [sept, $^3J_{H-H}$ = 6.3 Hz, 1 H, NC*<u>H</u>*(CH_3)$_2$], 2.15 (s, 3 H, Me^5), 2.03 (s, 3 H, Me^3), 1.98 [t, $^3J_{H-H}$ = 8.2 Hz, 3 H, $MgCH_2$C*<u>H</u>*$_3$], 1.24 [d, $^3J_{H-H}$ = 6.3 Hz, 3 H, NCH(C*<u>H</u>*$_3$)$_2$], 1.07 [d, $^3J_{H-H}$ = 6.3 Hz, 3 H, NCH(C*<u>H</u>*$_3$)$_2$], 0.31 [q, $^3J_{H-H}$ = 8.2 Hz, 2 H, MgC*<u>H</u>*$_2$$CH_3$], –0.23 (s, 6 H, $AlMe_2$). **^{13}C-{^{1}H}-NMR** (125 MHz, C_6D_6, 297 K), δ 164.2 (C^b), 149.2, 144.3 ($C^{3 \text{ or } 5}$), 106.4 (C^4), 76.5 (C^a), 46.5 [N*<u>C</u>*H(CH_3)$_2$], 46.4 [N*<u>C</u>*H(CH_3)$_2$], 25.6 [NCH(*<u>C</u>*H_3)$_2$], 24.6 [NCH(*<u>C</u>*H_3)$_2$], 24.6 ($MgCH_2$*<u>C</u>*H_3), 13.3 (Me^3), 13.0 (Me^5), –1.9 (Mg*<u>C</u>*H_2CH_3), –3.7 [*br*, $AlMe_2$].

Synthesis of complex [$AlMe_2$(pbpamd$^-$)Mg^{t}Bu{κ^1-O-(OC_4H_8O)}] (2)

The synthesis of complex **2a** was carried out in an identical manner to **1a** using Hpbpamd (1.00 g, 3.03 mmol), $AlMe_3$ (2.0 M in toluene) (1.51, 3.03 mmol) and ^{t}BuMgCl (1.0 M in tetrahydrofuran) (9.09 mL, 9.09 mmol). Yield: 70 %, 1.18 g. Anal.Calcd. (%) for $C_{28}H_{51}AlMgN_6O_2$: C, 60.59; H, 9.26; N, 15.14. Found: C, 60.40; H, 9.11; N, 15.09.

2a. **^{1}H-NMR** (500 MHz, C_6D_6, 297 K) δ 5.42 (s, 1 H, H^4), 5.34 (s, 1 H, $H^{4'}$), 4.25 [sept, $^3J_{H-H}$ = 6.3 Hz, 1 H, NC*H*(CH_3)$_2$], 3.81 [sept, $^3J_{H-H}$ = 6.3 Hz, 1 H, NC*H*(CH_3)$_2$], 3.37 (*br*, 8 H, OC_4H_8O), 2.07 (s, 3 H, Me^5), 1.96 (s, 3 H, $Me^{5'}$), 1.66 (s, 3 H, Me^3), 1.52 (s, 9 H, ^{t}Bu), 1.48 (s, 3 H, $Me^{3'}$), 1.30 [d, $^3J_{H-H}$ = 6.3 Hz, 3 H, NCH(C*H*$_3$)$_2$], 1.14 [d, $^3J_{H-H}$ = 6.3 Hz, 3 H, NCH(C*H*$_3$)$_2$], 1.08 [d, $^3J_{H-H}$ = 6.3 Hz, 3 H, NCH(C*H*$_3$)$_2$], 0.96 [d, $^3J_{H-H}$ = 6.3 Hz, 3 H, NCH(C*H*$_3$)$_2$], –0.04 [s, 3 H, Al(C*H*$_3$)$_2$], –0.13 [s, 3 H, Al(C*H*$_3$)$_2$]. **^{13}C-{^{1}H}-NMR** (125 MHz, C_6D_6, 297 K), δ 163.2 (C^b), 147.4, 145.8, 144.0, 142.0 ($C^{3,3'\text{ or }5,5'}$), 106.0 (C^4), 105.1 ($C^{4'}$), 93.6 (C^a), 69.0 ($C_4H_8O_2$), 50.7 [N*C*H(CH_3)$_2$], 46.3 [N*C*H(CH_3)$_2$], 35.8 [MgC(*C*H$_3$)$_3$], 26.7 [NCH(*C*H$_3$)$_2$], 25.6 [NCH(*C*H$_3$)$_2$], 25.0 [NCH(*C*H$_3$)$_2$], 24.6 [NCH(*C*H$_3$)$_2$], 14.3 (Me^3), 13.6 [Mg*C*(CH_3)$_3$], 13.0 ($Me^{3'}$), 10.8 (Me^5), 10.4 ($Me^{5'}$), –5.6 [*br*, $AlMe_2$], –7.9 [*br*, $AlMe_2$]. **2b**. **^{1}H-NMR** (500 MHz, C_6D_6, 297 K), δ 5.28 (s, 1 H, H^4), 3.87 [sept, $^3J_{H-H}$ = 6.2 Hz, 1 H, NC*H*(CH_3)$_2$], 3.75 [sept, $^3J_{H-H}$ = 6.2 Hz, 1 H, NC*H*(CH_3)$_2$], 2.13 (s, 3 H, Me^5), 2.07 (s, 3 H, Me^3), 1.64 (s, 9 H, ^{t}Bu), 1.22 [d, $^3J_{H-H}$ = 6.2 Hz, 3 H, NCH(C*H*$_3$)$_2$], 1.05 [d, $^3J_{H-H}$ = 6.2 Hz, 3 H, NCH(C*H*$_3$)$_2$], –0.25 (s, 6 H, $AlMe_2$). **^{13}C-{^{1}H}-NMR** (125 MHz, C_6D_6, 297 K), δ 165.0 (C^b), 149.3, 144.3 ($C^{3\text{ or }5}$), 106.8 (C^4), 76.3 (C^a), 46.9 [N*C*H(CH_3)$_2$], 46.6 [N*C*H(CH_3)$_2$], 35.0 [MgC(*C*H$_3$)$_3$], 25.6 [NCH(*C*H$_3$)$_2$], 25.2 [NCH(*C*H$_3$)$_2$], 13.8 [Mg*C*(CH_3)$_3$], 13.4 (Me^3), 13.1 (Me^5), –3.7 [*br*, $AlMe_2$].

3. Spectroscopic details

Figures S1–S2. ^{1}H and ^{13}C-^{1}H NMR spectra of complexes **1a**,**b**–**2a**,**b**.

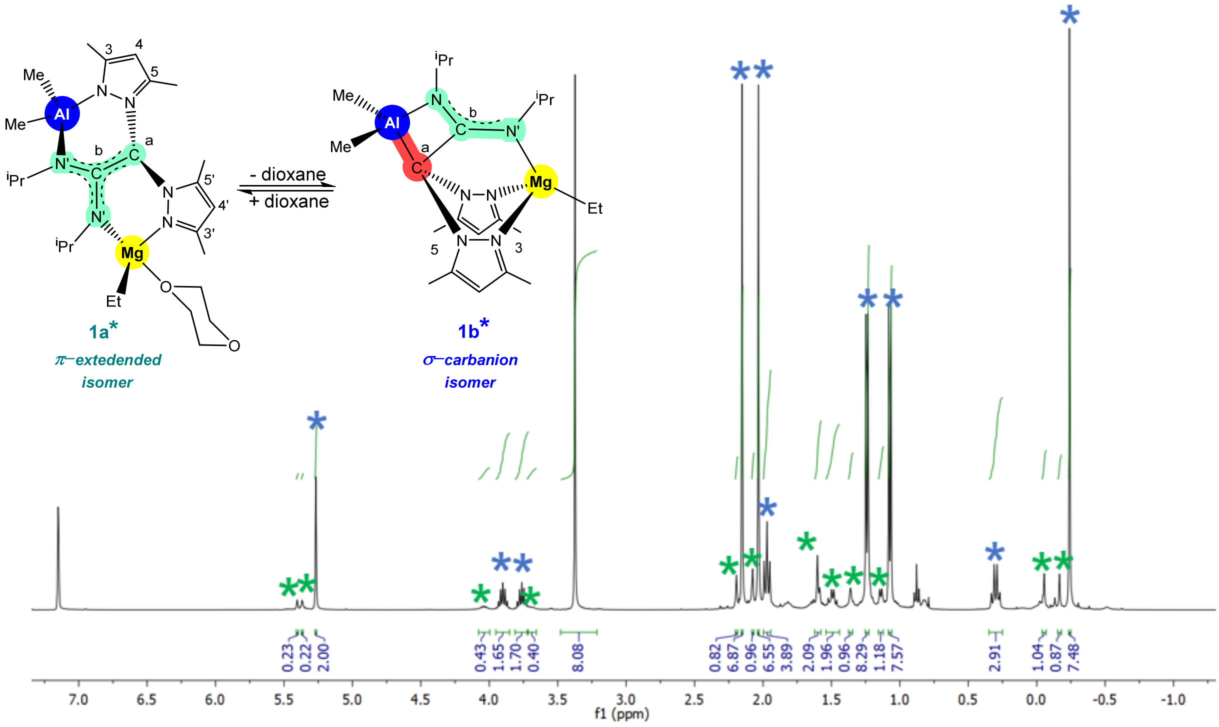

Figure S1a. ^{1}H-NMR spectrum (500 MHz, 297 K, C_6D_6) for complex [$AlMe_2(pbpamd^{-})MgEt\{\kappa^1\text{-}O\text{-}(OC_4H_8O)\}$] (**1**) in a ratio **1a:1b** 1:4.

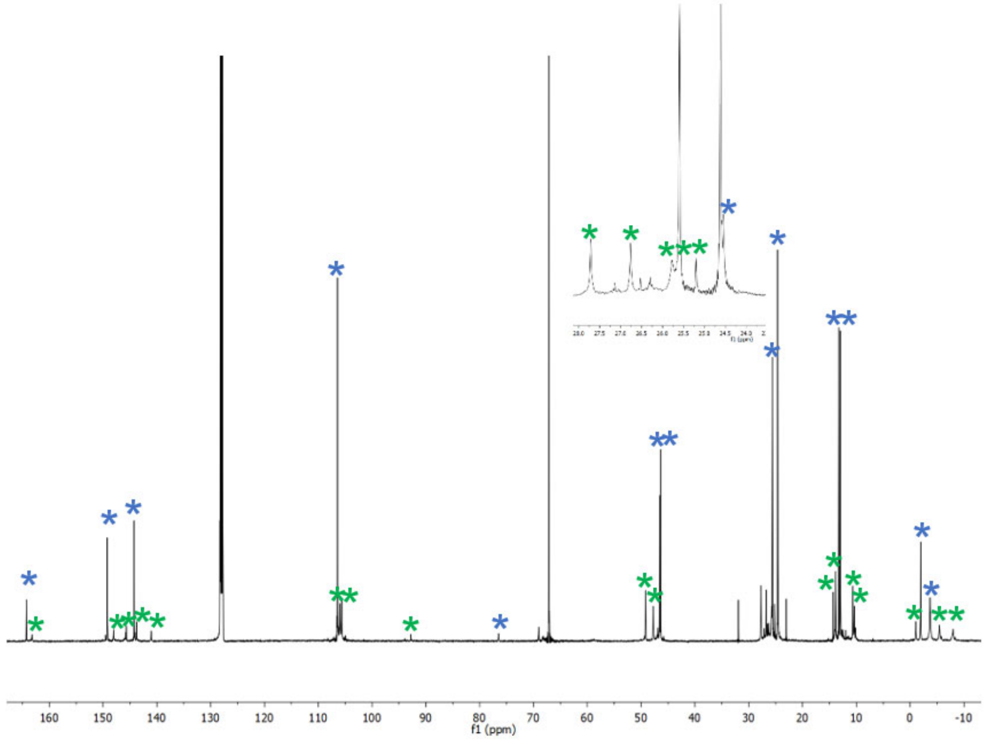

Figure S1b. ^{13}C-{^{1}H}-NMR spectrum (125 MHz, 297 K, C_6D_6) for complex [$AlMe_2(pbpamd^{-})MgEt\{\kappa^1\text{-}O\text{-}(OC_4H_8O)\}$] in a ratio **1a:1b** 1:4 (**1**).

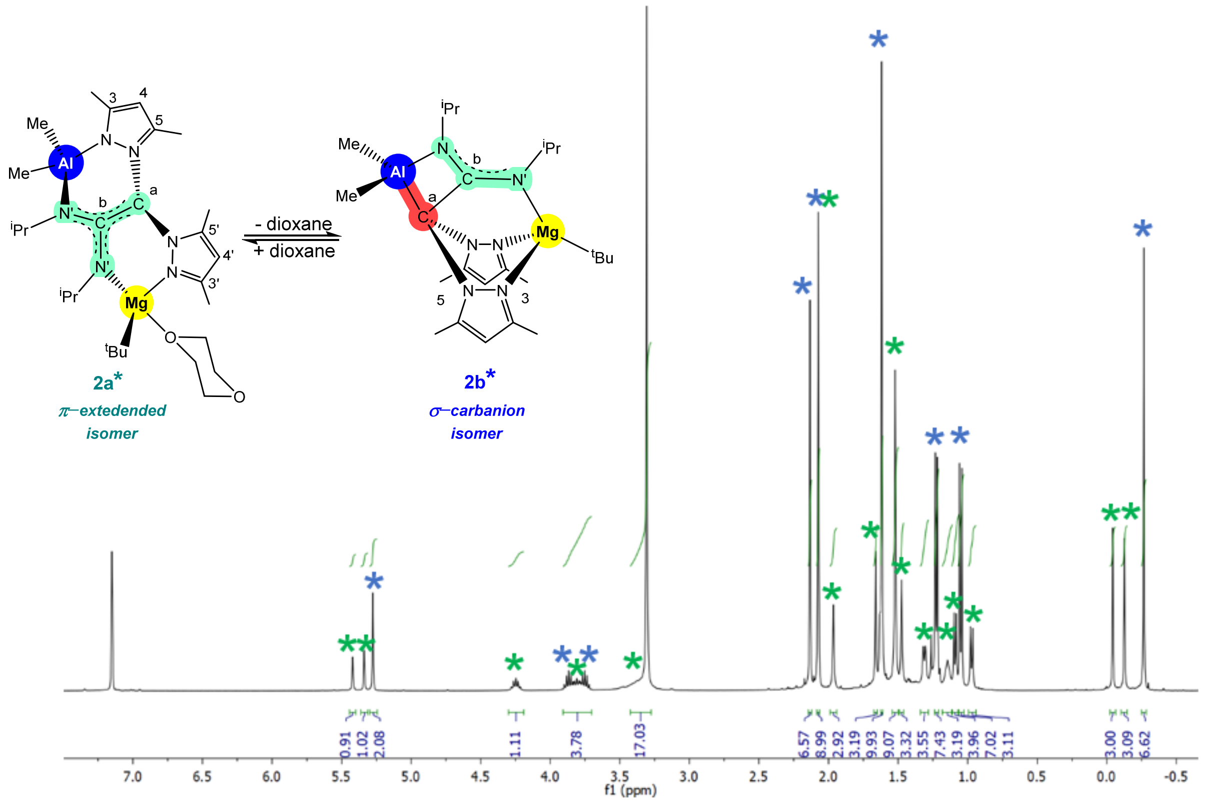

Figure S2a. ^{1}H-NMR spectrum (500 MHz, 297 K, C_6D_6) for complex [$AlMe_2$(pbpamd^{-})Mg^{t}Bu{κ^1-O-(OC_4H_8O)}] (**2**) in a ratio **2a:2b** 1:1.

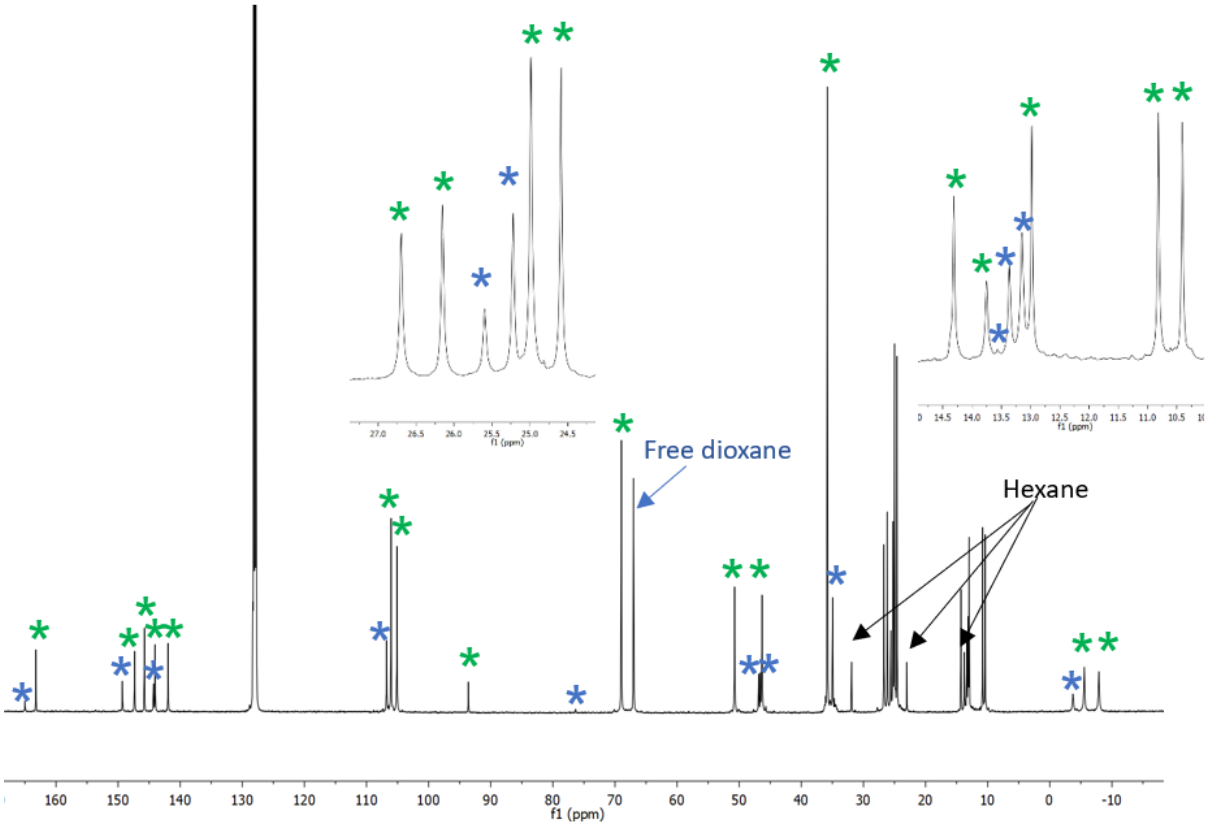

Figure S2b. ^{13}C-{^{1}H}-NMR spectrum (125 MHz, 297 K, C_6D_6) for complex [$AlMe_2$(pbpamd^{-})Mg^{t}Bu{κ^1-O-(OC_4H_8O)}] (**2**) in a ratio **2a:2b** 4:1.

4. Structural studies

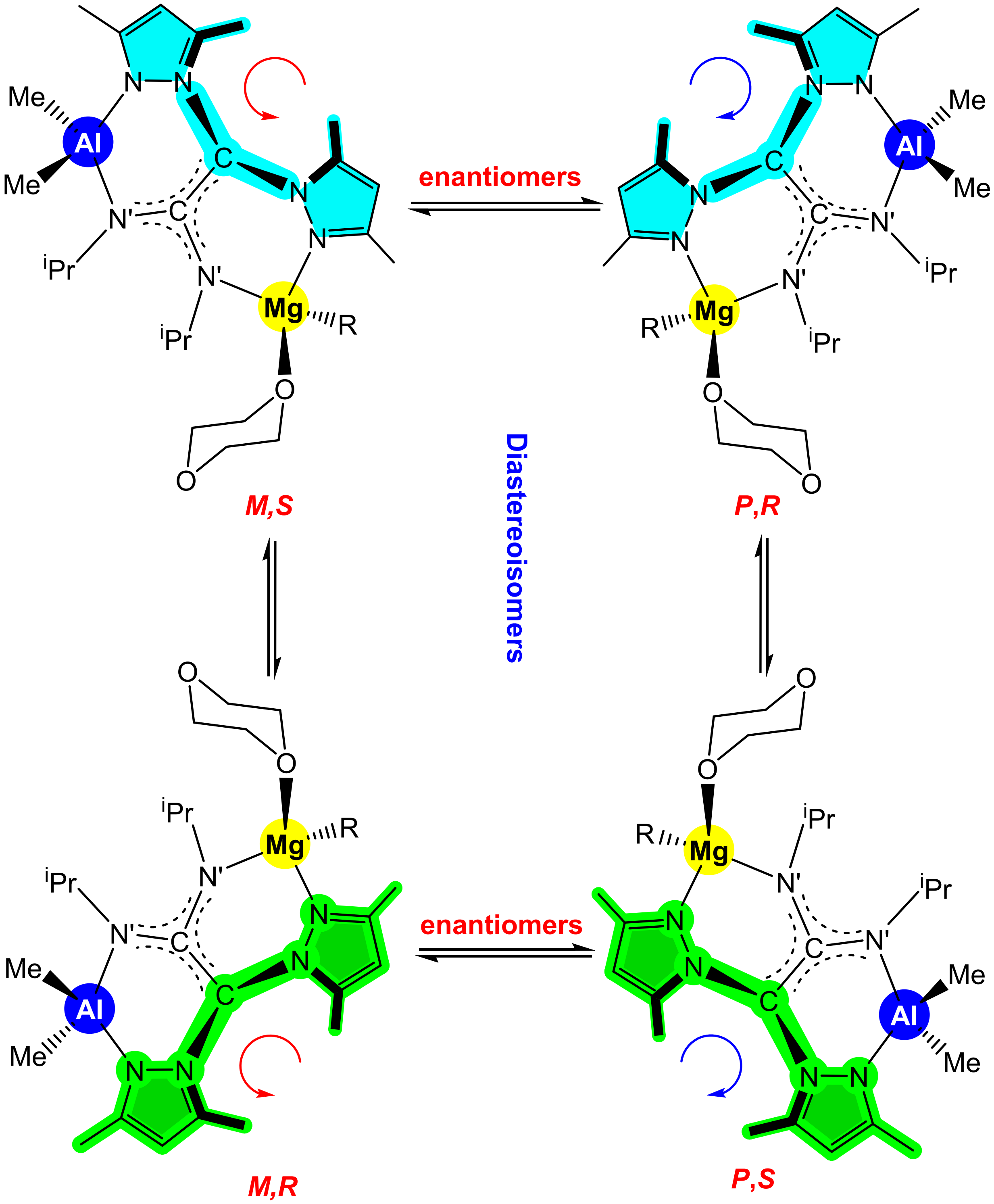

Figure S3. Two possible pairs of enantiomers for complexes [$AlMe_2$(pbpamd$^-$)MgR{κ^1-O-(OC_4H_8O)}] (R = Et **1a**, ^{t}Bu **2a**)

SUPPORTING INFORMATION TO

5. Dynamic behavior studies

Variable-temperature studies

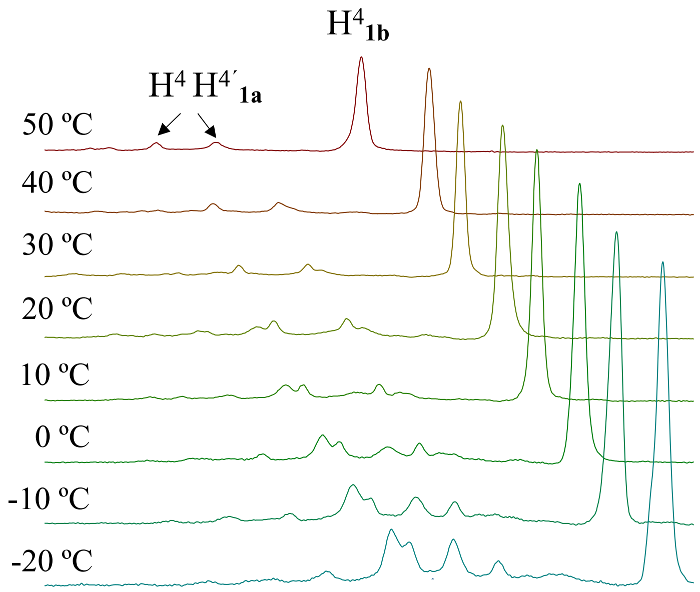

Figure S4. Variable-temperature ^{1}H NMR spectra in the region of the protons $H^{4,4'}$ for complex $[AlMe_2(pbpamd^-)MgEt\{\kappa^1\text{-}O\text{-}(OC_4H_8O)\}]$ (**1a**).

The free-energy value, $\Delta G^{\ddagger}$, was calculated by employing equation 1:[2]

$$\Delta G^{\neq} = aTc\left[9.972 + \log\left(\frac{T_c}{\Delta\nu}\right)\right] \qquad \textbf{\textit{(Eq. 1)}}$$

- $a = 1.914 \times 10^{-2}$ kJ/mol
- $T_c = 293.15$ K
- $\Delta\nu = 38$ Hz

$$\boldsymbol{\Delta G^{\neq}}_{\text{(exp)}} = \mathbf{60.93\ kJ/mol}$$

6. Dependence on the donor solvent concentration

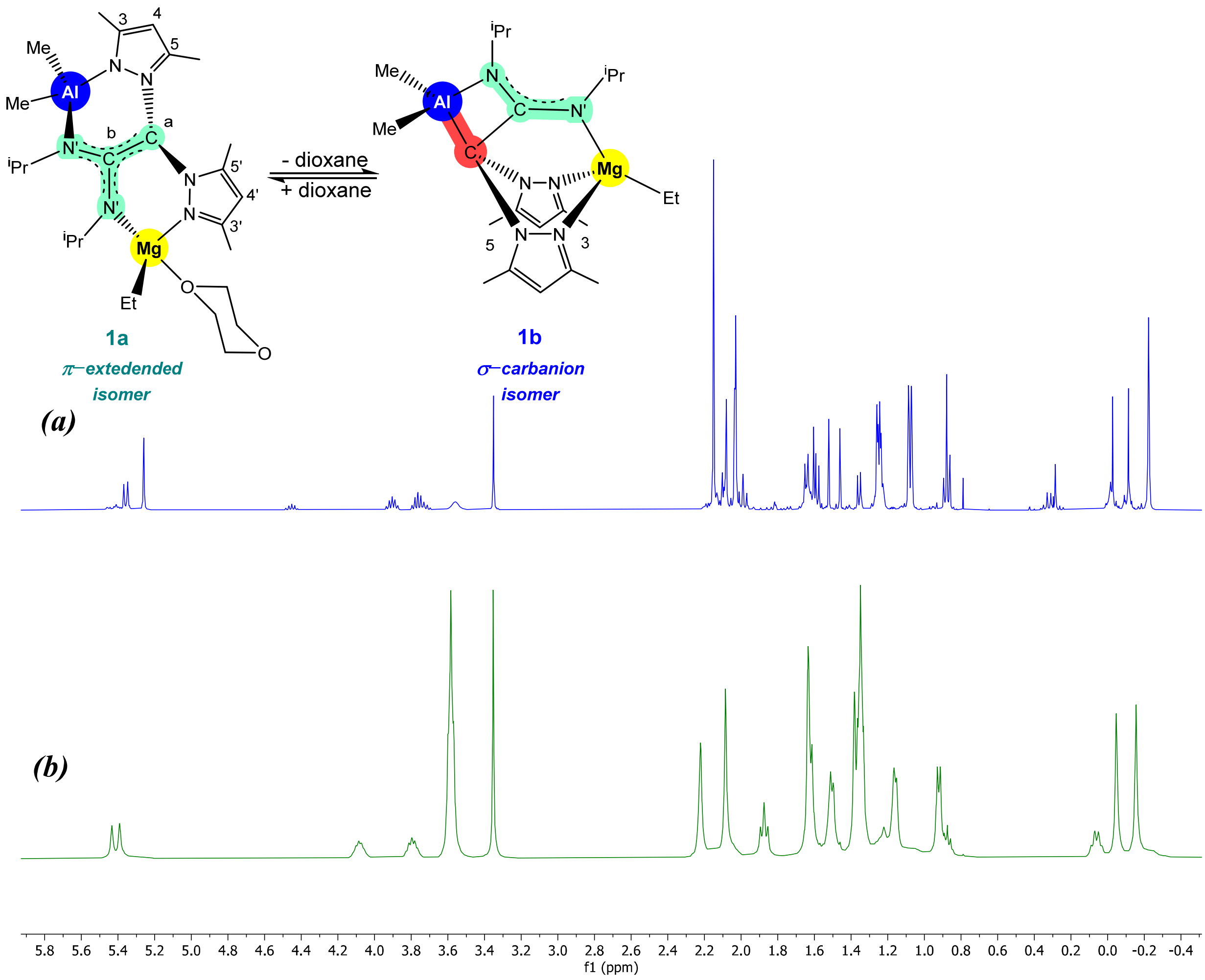

Figure S5. ^{1}H-NMR spectrum (500 MHz, 297 K, C_6D_6) (*a*) for complex **1** with [$AlMe_2$(pbpamd$^-$)MgEt{κ^1-O-(OC_4H_8O)}] (**1a**) and [MgEt(pbpamd$^-$)$AlMe_2$] (**1b**) in a 1:1 ratio. (*b*) for complex **1** in excess of tetrahydrofuran (C_4H_8O), showing a **1a'**(κ^1-OC_4H_8) and **1b** ratio of 1:0.

7. 1D and 2D ^{1}H NMR solution studies: selective 1D NOESY, DOSY and EXSY Experiments

Selective 1D NOESY Experiments

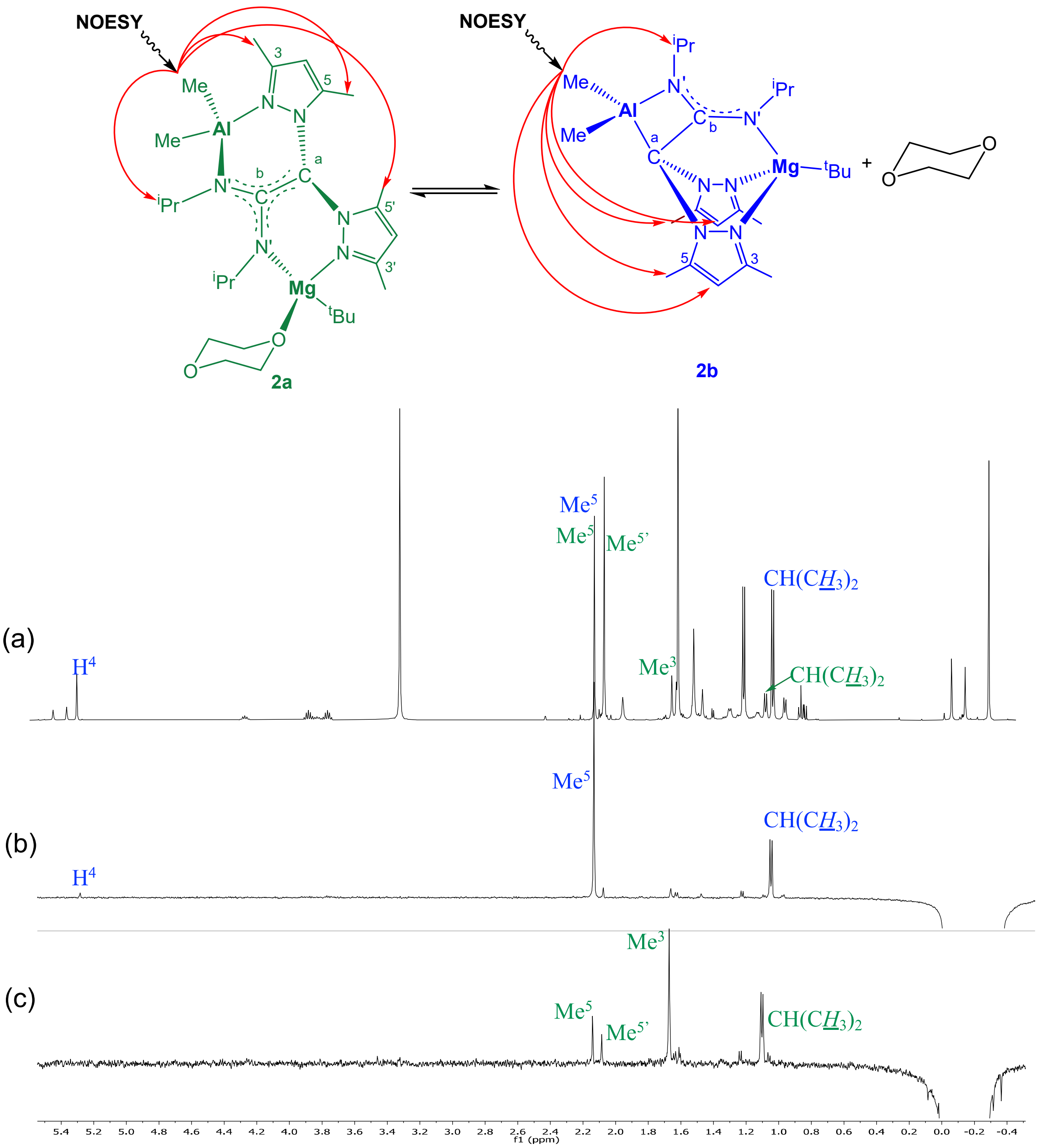

Figure S6. (*a*) ^{1}H NMR spectrum (500 MHz, 297 K, C_6D_6) for a mixture of [$AlMe_2$(pbpamd)$Mg^tBu\{\kappa^1$-O-(OC_4H_8O)}] (**2a**) and [Mg^tBu(pbpamd$^-$)$AlMe_2$] (**2b**), in a 1:2 ratio. (*b*) ^{1}H NOESY-1D responses on irradiating the signal of both methyl groups in $AlMe_2$ of **2b**. (*c*) ^{1}H NOESY-1D responses on irradiating the signals of both methyl groups in $AlMe_2$ of **2a**.

DOSY Experiment

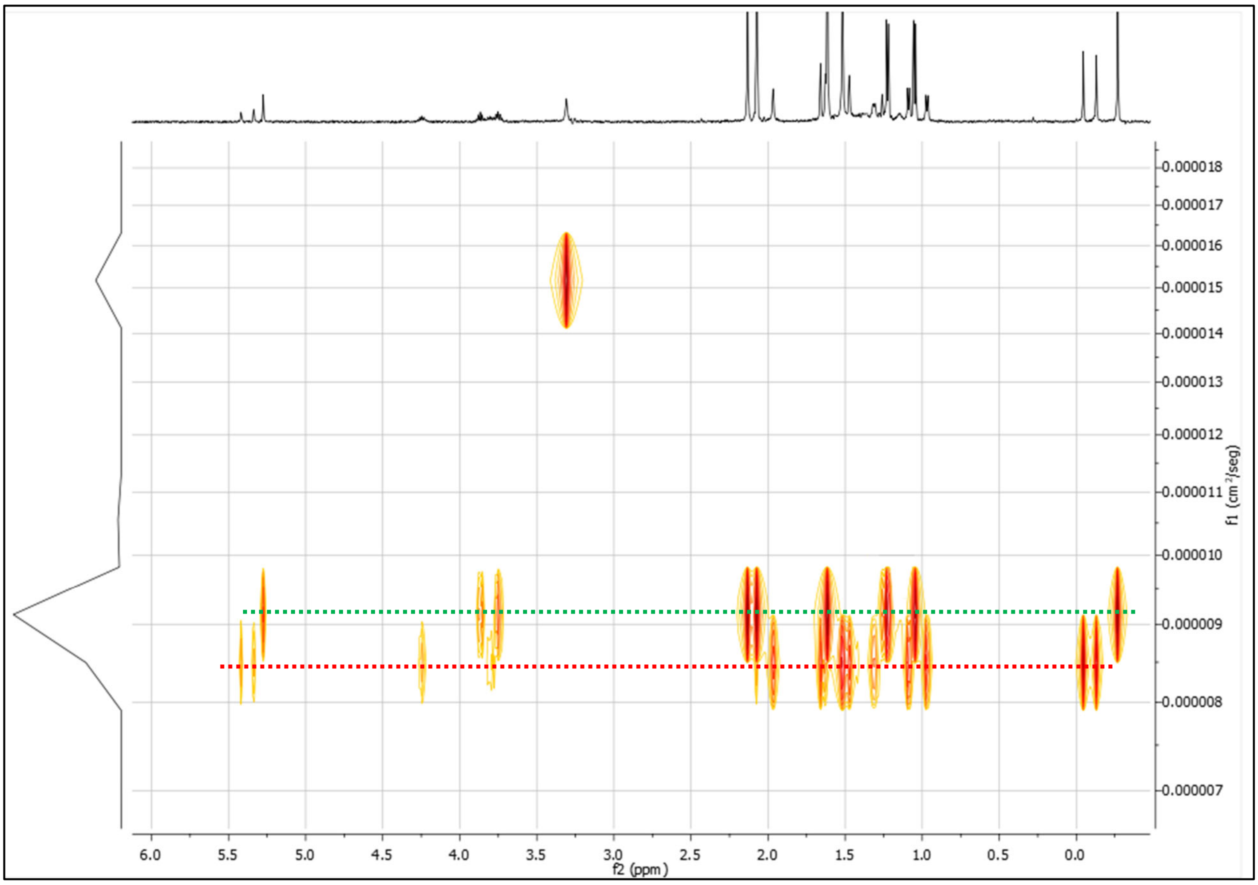

Figure S7. DOSY responses for a mixture of [AlMe$_2$(pbpamd)Mg^{t}Bu{κ^1-O-(OC$_4$H$_8$O)}] (**2a**) and [Mg^{t}Bu(pbpamd$^-$)AlMe$_2$] (**2b**), in a 1:1 ratio.

2D EXSY Experiment

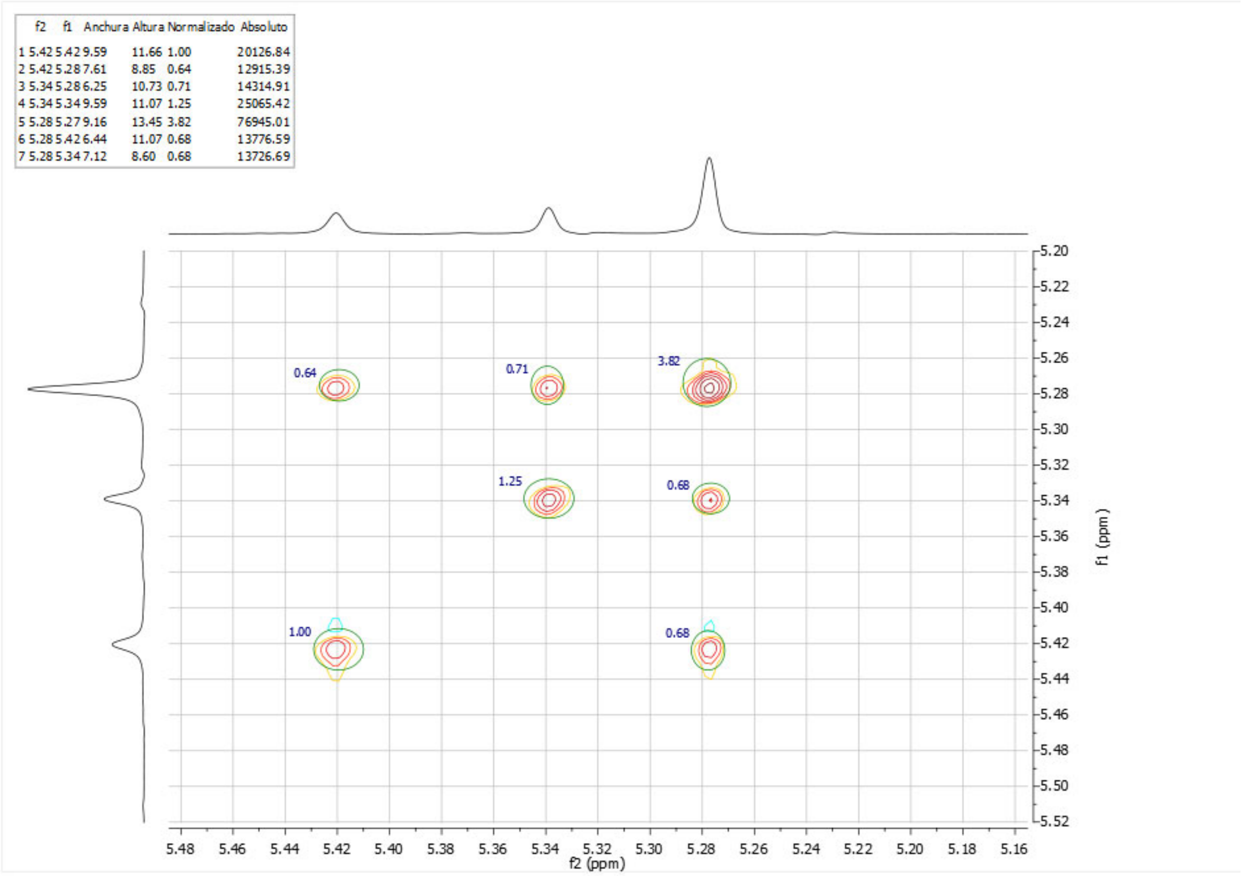

Figure S8. 2D EXSY responses in the H^4 area for a mixture of [AlMe$_2$(pbpamd)Mg^{t}Bu{κ^1-O-(OC$_4$H$_8$O)}] (**2a**) and [Mg^{t}Bu(pbpamd$^-$)AlMe$_2$] (**2b**) in a 1:1 ratio.

SUPPORTING INFORMATION TO

8. DFT calculations for complex 2

Computational Details

All the calculations reported in this paper were performed with the Gaussian 16 suite of programs.[3] Electron correlation was partially taken into account using the hybrid functional usually denoted as B3LYP[4] in conjunction with the D3 dispersion correction suggested by Grimme et al.[5] using the double-ζ quality def2-SVP[6] basis set for all atoms. All species were characterized by frequency calculations, and have positive definite Hessian matrices. Solvent effects (solvent = toluene) were taken into account using the Polarizable Continuum Model (PCM)[7] during the geometry optimizations. Energy refinements were carried out by means of single-point calculations at the same DFT level using the much larger triple-ζ basis set def2-TZVP.[6] This level is denoted PCM(toluene)-B3LYP-D3/def2-TZVP//PCM(toluene)-B3LYP–D3/def2-SVP. Chemical shifts were computed using the using the gauge invariant atomic orbital (GIAO)[8] method at the PCM-B3LYP-D3/def2-SVP level with the optimized PCM(toluene)-B3LYP–D3/def2-SVP geometries.

Thermodynamic stability study of the possible reorganization products of complex 2a in toluene.

Herein we present the input coordinates for complex **2a** and **2b**, taken into the account for the latter one the two different coordination arrangements of the scorpionate ligand, namely apical σ-C(sp^3)-Al mode and σ-C(sp^3)-Mg mode, respectively. Gibbs free energies (ΔG) were also evaluated by frequency calculations (Table S1).

Table S1. Gibbs free energies values calculated at 298.15 K of optimized structures at the PCM(toluene)-B3LYP-D3/def2-TZVP//PCM(toluene)-B3LYP–D3/def2-SVP level for the different ligand arrangements in heterodinuclear aluminium-magnesium complexes.

**Complex**	Disposition	ΔG (Ha)	ΔΔG (Ha)[a]	ΔΔG (kcal/mol)
**2a**	Al(*π*-C_2N_2(*sp*^{2}))Mg	-2020.0344144		
**2b**	*σ*-C(*sp*^{3})-Al	-1712.2075346	0.0106348	**6.7**
	σ-C(*sp*^{3})-Mg	-1712.1785776	0.0387108	**24.3**
**dioxane**	–	-307.79641		

[a] ΔΔG = ΔG (Al(*π*-C_2N_2(*sp*^{2})Mg) – [ΔG (σ-C(*sp*^{3})-M) + ΔG (dioxane)] (M = Al or Mg)

SUPPORTING INFORMATION TO
Input Coordinates

[AlMe$_2$(pbpamd$^-$)Mg^{t}Bu{κ^1-O-(OC$_4$H$_8$O)}], 2a-Al(π-C$_2$N$_2$(sp^2))Mg

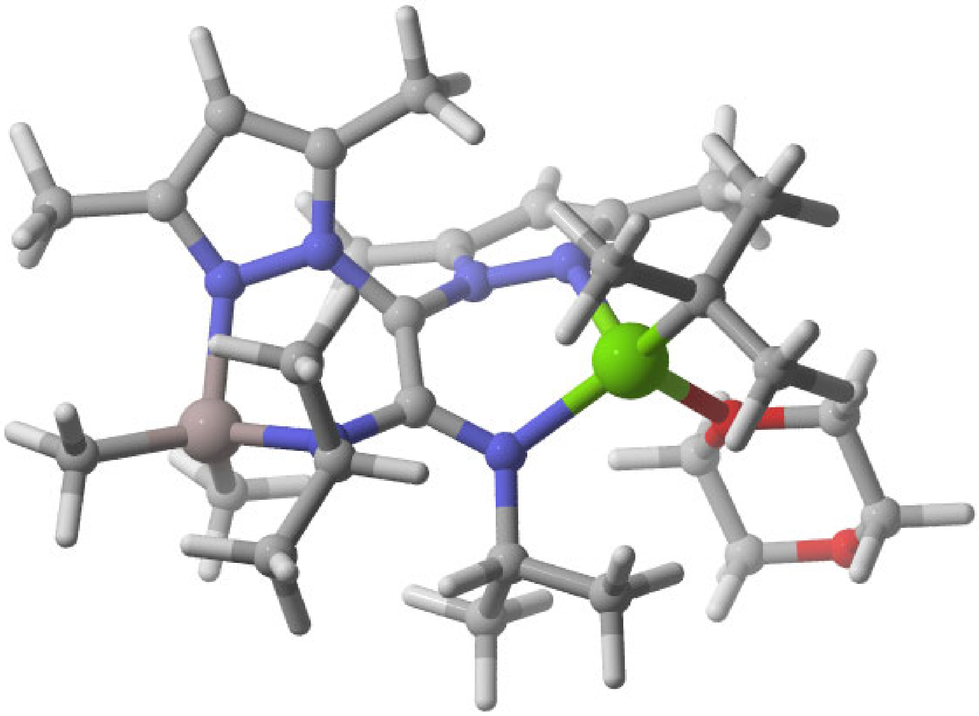

ZPE at 298 K: -2017.392521 Ha

Gibbs Energy at 298 K: -2017.469792 Ha

HF = -2020.0344144 Ha

Gibbs Energy at 298 K (TZ): -2020.111685 Ha

Atomic Number	*X*	*Y*	*Z*
12	1.838087	-0.105401	0.812076
13	-3.420215	-1.095749	-1.106735
7	1.025269	1.735377	0.062967
7	-0.172111	1.593055	-0.579073
7	-3.541177	0.726687	-0.263203
7	-2.351271	1.149152	0.259273
7	0.432896	-1.230306	-0.187224
7	-1.950248	-1.617917	-0.031442
8	3.479409	0.05845	-0.48502
8	5.548276	-0.551059	-2.26918
6	-0.368331	2.616078	-1.460159

6	0.746977	3.445716	-1.369075
1	0.920004	4.363015	-1.927046
6	1.596297	2.847934	-0.415781
6	-1.609599	2.736651	-2.276903
1	-1.904985	1.7601	-2.686789
1	-1.453757	3.445503	-3.101283
1	-2.454801	3.09769	-1.667337
6	2.944159	3.295752	0.055321
1	2.93396	4.370624	0.29151
1	3.715744	3.142359	-0.716829
1	3.244823	2.747947	0.958699
6	-2.557257	2.155926	1.14926
6	-3.932234	2.393403	1.18122
1	-4.448529	3.137064	1.783385
6	-4.512825	1.463737	0.303898
6	-1.44429	2.807736	1.896427
1	-0.737343	2.054703	2.276497
1	-1.846373	3.382972	2.740871
1	-0.867904	3.492768	1.253593
6	-5.961363	1.237037	0.01094
1	-6.133612	1.080052	-1.064151
1	-6.553707	2.102276	0.339401
1	-6.336699	0.344413	0.535598
6	-1.1135	0.604178	-0.190736
6	-0.843934	-0.782061	-0.137743
6	0.678653	-2.580015	-0.71371
1	-0.112929	-3.276685	-0.400857
6	0.669189	-2.578811	-2.249226
1	-0.2959	-2.205891	-2.622693
1	0.82533	-3.593951	-2.651916
1	1.462818	-1.927994	-2.649368
6	1.993596	-3.126861	-0.152757
1	2.846021	-2.486243	-0.432929
1	2.20013	-4.139327	-0.532953
1	1.957589	-3.174631	0.947345

6	-1.954669	-2.556551	1.10676
1	-0.909528	-2.72755	1.414655
6	-2.668791	-1.925166	2.313681
1	-2.195659	-0.965836	2.576365
1	-2.625692	-2.582774	3.198313
1	-3.728509	-1.729686	2.081703
6	-2.56057	-3.914874	0.74306
1	-3.629967	-3.822766	0.502456
1	-2.467206	-4.620446	1.584496
1	-2.053683	-4.351136	-0.132061
6	-2.919489	-0.852131	-3.011088
1	-1.885854	-0.484236	-3.131719
1	-3.58377	-0.135915	-3.529887
1	-2.987032	-1.804239	-3.569258
6	-5.156991	-1.998226	-0.784009
1	-5.13722	-3.036632	-1.161766
1	-5.974641	-1.490041	-1.326157
1	-5.454205	-2.04876	0.278254
6	2.398108	-0.233407	2.884138
6	1.062579	-0.476197	3.605341
1	0.591626	-1.426663	3.296626
1	0.325804	0.321932	3.398438
1	1.179025	-0.518125	4.713971
6	2.986081	1.097172	3.367015
1	3.166075	1.109071	4.468119
1	2.315947	1.94852	3.148024
1	3.95871	1.321832	2.890283
6	3.358937	-1.371061	3.241617
1	4.351046	-1.253371	2.767567
1	2.971155	-2.356378	2.927571
1	3.544419	-1.437314	4.33981
6	4.851747	0.143947	-0.064087
1	4.865535	-0.102743	1.005569
1	5.20052	1.181804	-0.204673
6	5.695672	-0.816728	-0.885645

1	6.760205	-0.695232	-0.63476
1	5.398411	-1.859415	-0.654384
6	4.196308	-0.671636	-2.677355
1	3.840625	-1.710576	-2.533909
1	4.150346	-0.435794	-3.751148
6	3.310281	0.286148	-1.896796
1	3.582923	1.331084	-2.121582
1	2.24304	0.13657	-2.108149

SUPPORTING INFORMATION TO
[$Me_2Al(\kappa^2$-C,N';κ^3-NNN'-pbpamd$^-$)Mg^tBu], 2b-σ-C(sp^3)-Al

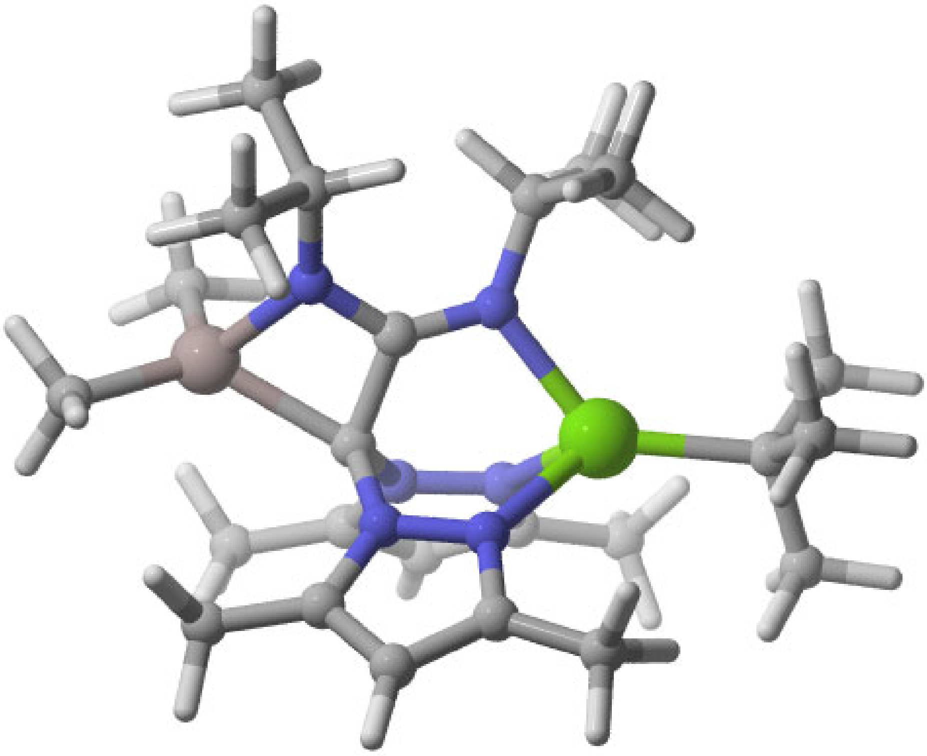

ZPE at 298 K: -1710.035512 Ha

Gibbs Energy at 298 K: -1710.104919 Ha

HF = -1712.2075346 Ha

Gibbs Energy at 298 K (TZ): -1712.276942 Ha

Atomic Number	*X*	*Y*	*Z*
6	0.984686	-1.34228	2.777454
6	-0.118178	-2.12734	3.162414
1	-0.214005	-2.738878	4.056543
6	-1.071424	-1.972533	2.161438
6	2.277699	-1.138162	3.501455
1	3.133225	-1.212709	2.815294
1	2.398677	-1.887878	4.29533
1	2.316909	-0.139767	3.966665
6	1.475005	-2.048364	-2.203809
6	0.409162	-2.889173	-2.584914
1	0.43194	-3.70608	-3.302236

6	-0.691107	-2.472683	-1.847053
6	2.895943	-2.062871	-2.672046
1	2.948487	-2.257932	-3.753414
1	3.473663	-2.852233	-2.163574
1	3.386038	-1.101608	-2.464541
6	-2.049447	-3.090009	-1.811243
1	-2.297927	-3.457264	-0.804416
1	-2.073442	-3.948654	-2.495434
1	-2.835261	-2.389429	-2.12162
6	-1.035485	-0.669561	-0.071335
6	-0.884607	0.885468	-0.13132
6	0.399937	2.834992	-0.877092
1	-0.588048	3.287725	-1.04076
6	1.173173	3.691174	0.13069
1	0.660602	3.722408	1.104936
1	1.293192	4.72393	-0.233034
1	2.178333	3.271167	0.295823
6	1.104471	2.802745	-2.240774
1	2.10354	2.346541	-2.154243
1	1.22923	3.820295	-2.645325
1	0.521326	2.207898	-2.960255
6	-2.444507	2.740387	0.555917
1	-1.522995	3.302812	0.765115
6	-3.233761	2.658417	1.870374
1	-4.175329	2.104636	1.725592
1	-3.480934	3.666045	2.241509
1	-2.647702	2.135191	2.641596
6	-3.246769	3.481019	-0.521607
1	-2.692124	3.534417	-1.470844
1	-3.485853	4.506827	-0.198975
1	-4.196429	2.957418	-0.720329
6	-2.418702	-2.60722	2.090492
1	-3.188651	-1.96369	2.539036
1	-2.403734	-3.554066	2.64804
1	-2.729451	-2.814016	1.060366

7	-0.543204	-1.112286	1.244541
7	0.72402	-0.744362	1.611582
7	-0.283007	-1.407686	-1.091696
7	1.048389	-1.175577	-1.290947
7	0.265989	1.454331	-0.401013
7	-2.105575	1.385099	0.130593
12	1.917609	0.246305	0.062023
13	-3.088817	-0.152757	-0.421777
6	-3.43304	0.008371	-2.376736
1	-2.510531	-0.035759	-2.983033
1	-4.142628	-0.725913	-2.797876
1	-3.877778	1.005222	-2.557411
6	-4.670677	-0.662653	0.652627
1	-4.972027	-1.717726	0.528451
1	-4.572876	-0.469174	1.733669
1	-5.521087	-0.052649	0.29342
6	4.0374	0.659662	0.26068
6	4.567088	1.43181	-0.955909
1	5.667426	1.600903	-0.893887
1	4.384222	0.901183	-1.908151
1	4.102648	2.428618	-1.050217
6	4.811557	-0.663201	0.368851
1	5.907732	-0.495454	0.484998
1	4.494963	-1.266912	1.238037
1	4.683291	-1.299865	-0.522527
6	4.324782	1.491985	1.519141
1	3.993218	0.987127	2.442261
1	5.41426	1.69531	1.64215
1	3.823738	2.47565	1.494506

SUPPORTING INFORMATION TO
[$Me_2Al(\kappa^2$-N,N';κ^2-C,N'-pbpamd$^-$)Mg^{t}Bu], 2b-σ-C(sp^3)-Mg

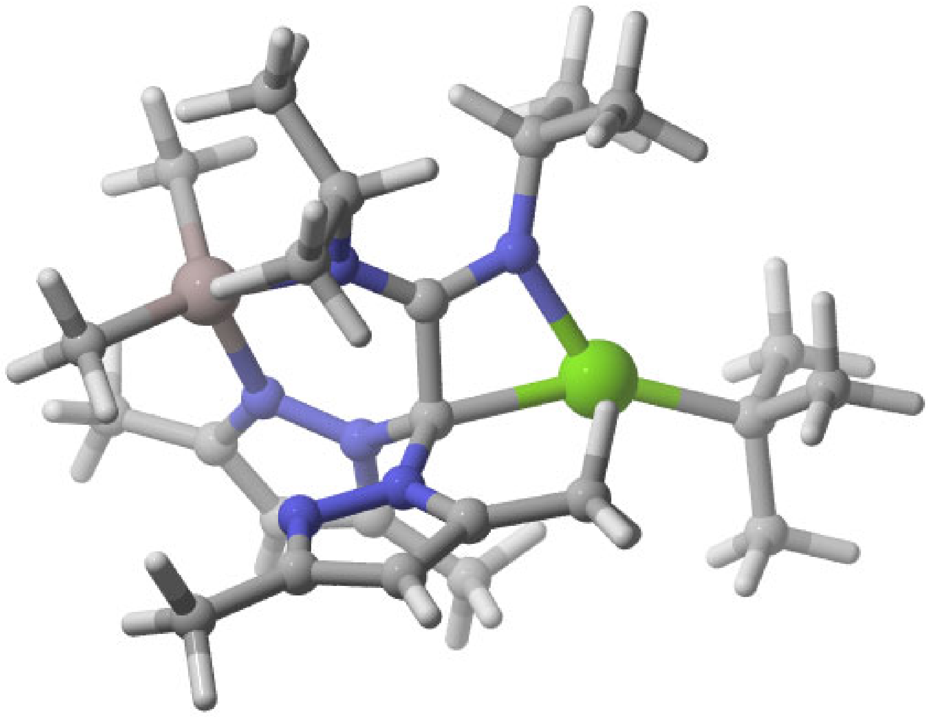

ZPE at 298 K: -1710.004453 Ha

Gibbs Energy at 298 K: -1710.074741 Ha

HF = -1712.1785776 Ha

Gibbs Energy at 298 K (TZ): -1712.248866 Ha

Atomic Number	*X*	*Y*	*Z*
6	2.035336	-1.871751	2.38844
6	1.076995	-2.899481	2.423649
1	1.114323	-3.809031	3.017707
6	0.068701	-2.517809	1.545506
6	3.309286	-1.770257	3.165852
1	4.156054	-1.502161	2.516399
1	3.533414	-2.730914	3.648789
1	3.234901	-0.999285	3.948729
6	1.876744	-1.723499	-2.678556
6	0.716849	-1.47409	-3.455729
1	0.592731	-1.584806	-4.531174
6	-0.254244	-1.072908	-2.547914

6	3.232474	-2.161817	-3.141975
1	3.806755	-1.311678	-3.548701
1	3.160552	-2.920837	-3.936688
1	3.806151	-2.58122	-2.303222
6	-1.675948	-0.681774	-2.785579
1	-2.383973	-1.328007	-2.237017
1	-1.916902	-0.782234	-3.852397
1	-1.873674	0.368057	-2.50754
6	-0.229096	-0.614411	-0.059146
6	-0.047242	0.915616	0.041931
6	1.368769	2.637249	-1.001722
1	0.382884	3.058065	-1.244666
6	2.199257	3.735088	-0.331047
1	1.756283	4.053025	0.624534
1	2.273617	4.613409	-0.991239
1	3.226115	3.39042	-0.126878
6	2.003408	2.188272	-2.327934
1	3.0039	1.762597	-2.155418
1	2.102913	3.03954	-3.021971
1	1.389829	1.410012	-2.806915
6	-1.376379	2.923568	0.607524
1	-0.397922	3.415842	0.720939
6	-2.086707	2.987909	1.966419
1	-3.070517	2.490416	1.912553
1	-2.249837	4.031729	2.277871
1	-1.487902	2.484659	2.74145
6	-2.187711	3.648191	-0.472012
1	-1.686819	3.601383	-1.451288
1	-2.3365	4.707738	-0.210618
1	-3.179931	3.178835	-0.579751
6	-1.183247	-3.230759	1.160579
1	-2.082301	-2.69218	1.503919
1	-1.200606	-4.229258	1.616577
1	-1.261991	-3.339001	0.068738
7	0.418958	-1.311429	1.030092

7	1.635548	-0.926142	1.524719
7	0.341526	-1.095015	-1.315905
7	1.634735	-1.479446	-1.393225
7	1.176913	1.442885	-0.150277
7	-1.205901	1.517118	0.256308
13	2.605186	0.703316	0.906599
12	-2.406429	-0.153867	0.22728
6	-4.490826	-0.628691	0.217533
6	-5.148723	-0.202944	1.537154
1	-6.249684	-0.372167	1.52455
1	-4.751567	-0.765162	2.401402
1	-4.997423	0.870813	1.750091
6	-5.11166	0.180915	-0.932155
1	-4.684975	-0.091783	-1.914253
1	-6.210898	0.017559	-1.006517
1	-4.962771	1.268616	-0.803664
6	-4.753788	-2.119764	-0.022725
1	-5.842768	-2.341002	-0.097667
1	-4.293036	-2.479728	-0.960761
1	-4.358807	-2.750481	0.793061
6	4.287997	0.286689	-0.039643
1	4.987375	-0.305074	0.5788
1	4.102612	-0.280705	-0.964853
1	4.82778	1.212869	-0.311311
6	2.791557	1.777939	2.572475
1	1.817833	1.928457	3.074894
1	3.466457	1.309081	3.311522
1	3.204786	2.782211	2.371296

SUPPORTING INFORMATION TO
$C_6H_4O_2$, dioxane

ZPE at 298 K: -307.320319 Ha

Gibbs Energy at 298 K: -307.348018 Ha

HF = -307.79641 Ha

Gibbs Energy at 298 K (TZ): -307.824109 Ha

Atomic Number	*X*	*Y*	*Z*
8	-0.225581	1.39023	0
8	0.225581	-1.39023	0
6	-0.225581	-0.728471	1.170428
1	-1.333342	-0.7669	1.231451
1	0.190232	-1.268157	2.035703
6	-0.225581	-0.728471	-1.170428
1	0.190232	-1.268157	-2.035703
1	-1.333342	-0.7669	-1.231451
6	0.225581	0.728471	1.170428
1	1.333342	0.7669	1.231451
1	-0.190232	1.268157	2.035703
6	0.225581	0.728471	-1.170428
1	1.333342	0.7669	-1.231451
1	-0.190232	1.268157	-2.035703

Expanded ^{13}C-{^{1}H}-NMR spectra of complex [$AlMe_2$(pbpamd$^-$)Mg^{t}Bu{κ^1-O-(OC_4H_8O)}] (2)

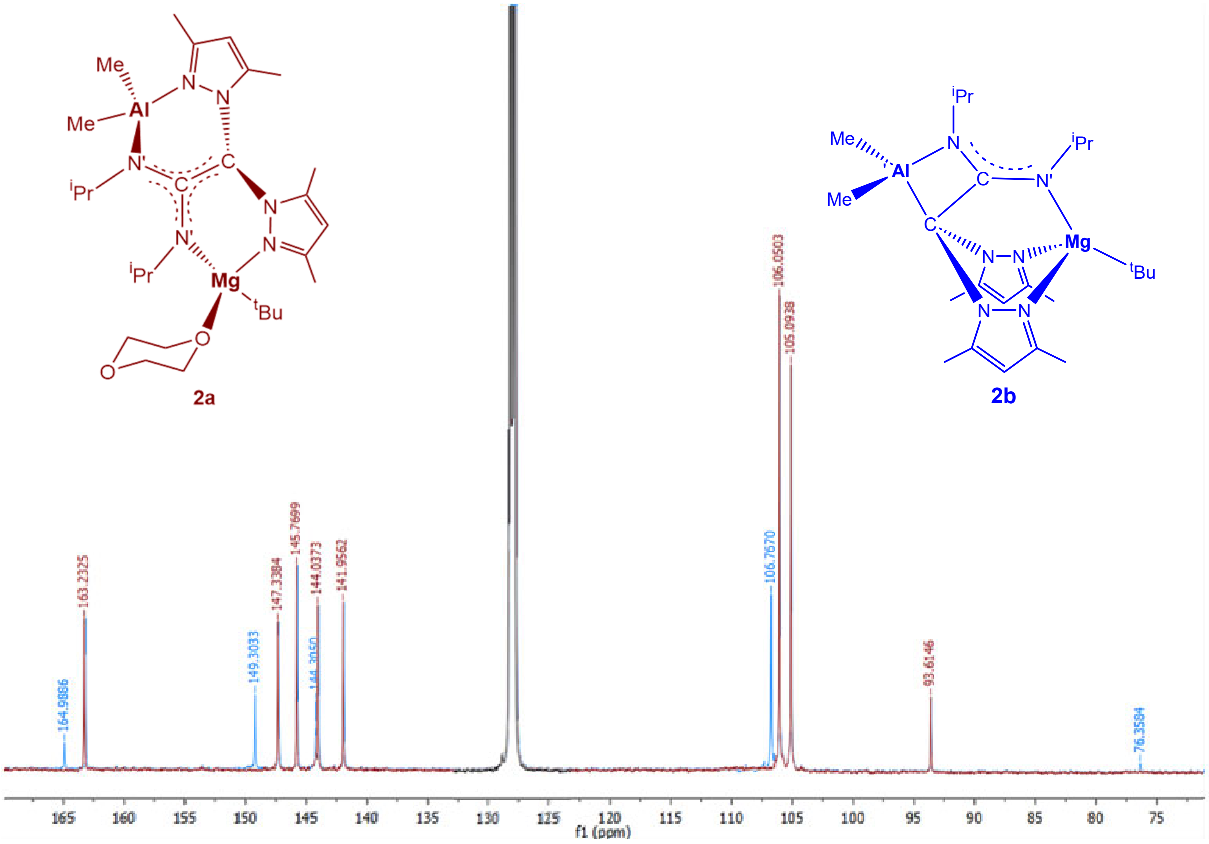

Figure S9. Expanded area of the experimental $^{13}C\{^1H\}$-NMR spectrum of complex **2** including signals of complexes **2a** (red) and **2b** (blue)

Table S2. Experimental and Computed $^{13}C\{^1H\}$-NMR chemical shifts for C^a and C^b atoms in complexes **2a**, **2b** and hypothetical **2b-Mg**(1)

	Experimental (ppm)		Computed (ppm)	
**Complex**	C^b	C^a	C^b	C^a
**2a**	163.2	93.6	160.8	92.9
**2b**	165.0	76.3	162.7	75.2
**2b-Mg**	-	-	172.5	76.6

(1)Hypothetical disposition of the complex where the heteroscorpionate ligand is coordinated as σ-carbanion to the magnesium center.

Table S3. Experimental and Computed $^{13}C\{^1H\}$-NMR chemical shifts differences in ppm between **2a** and **2b** or hypothetical **2b-Mg**(1) for C^a and C^b atoms.

	Experimental (ppm)		Computed (ppm)			
Diff. (ppm)	C^b	C^a	C^b	C^a	$\Delta C^{b(2)}$	$\Delta C^{a(2)}$
**2a-2b**	-1.8	17.3	-1.9	17.7	0.10	0.4
**2a-2b-Mg**	-	-	-11.7	16.3	1.0	9.9

(1)Hypothetical disposition of the complex where the heteroscorpionate ligand is coordinated as σ-carbanion to the magnesium center; (2) ABS($C^i_{comp.}$ - $C^i_{exp.}$) (i = a or b)

9. X-Ray diffraction studies: Crystallographic Structure Determination for Complex 2a.

Details for crystallographic studies and structural refinement

Crystals suitable for X-ray diffraction were obtained for **2a**. The crystal evaluation and data collection were performed on a Bruker X8 APEX II CCD-based diffractometer with MoKα (λ= 0.71073 Å) radiation. The initial cell constants were obtained from three series of scans at different starting angles. The reflections were successfully indexed by an automated indexing routine built in the SAINT program.[9] The absorption correction was based on fitting a function to the empirical transmission surface as sampled by multiple equivalent measurements.[10] A successful solution by the direct methods[11] provided most non-hydrogen atoms from the E-map. The remaining non-hydrogen atoms were located in an alternating series of least-squares cycles and difference Fourier maps. All non-hydrogen atoms were refined with anisotropic displacement coefficients unless specified otherwise. All hydrogen atoms were included in the structure factor calculation at idealized positions and were allowed to ride on the neighboring atoms with relative isotropic displacement coefficients.

SUPPORTING INFORMATION TO

Table S4. Crystal data and structure refinement for **2a**.

	**2a**
Empirical formula	$C_{28}H_{51}AlMgN_6O_2$
Formula weight	555.03
Temperature (K)	100(2)
Wavelength (Å)	0.71073
Crystal system	Monoclinic
Space group	*P 2₁/n*
a(Å)	10.2254(4)
b(Å)	22.8928(9)
c(Å)	13.9941(5)
α(°)	90
β(°)	96.577(2)
γ(°)	90
Volume(Å^3)	3254.3(2)
Z	4
Density (calculated) (g/cm^3)	1.133
Absorption coefficient (mm^{-1})	0.114
F(000)	1208
Crystal size (mm^3)	0.38 x 0.22 x 0.21
Index ranges	-12 ≤ h ≤ 12<br>-27 ≤ k ≤ 27<br>-16 ≤ l ≤ 16
Reflections collected	62565
Independent reflections	5956 [R(int) = 0.0455]
Data / restraints / parameters	5956 / 0 / 356
Goodness-of-fit on F^2	1.027
Final R indices [$I>2\sigma(I)$]	R1 = 0.0387<br>wR2 = 0.0971
Largest diff. peak / hole (e.Å^{-3})	0.298 / -0.454

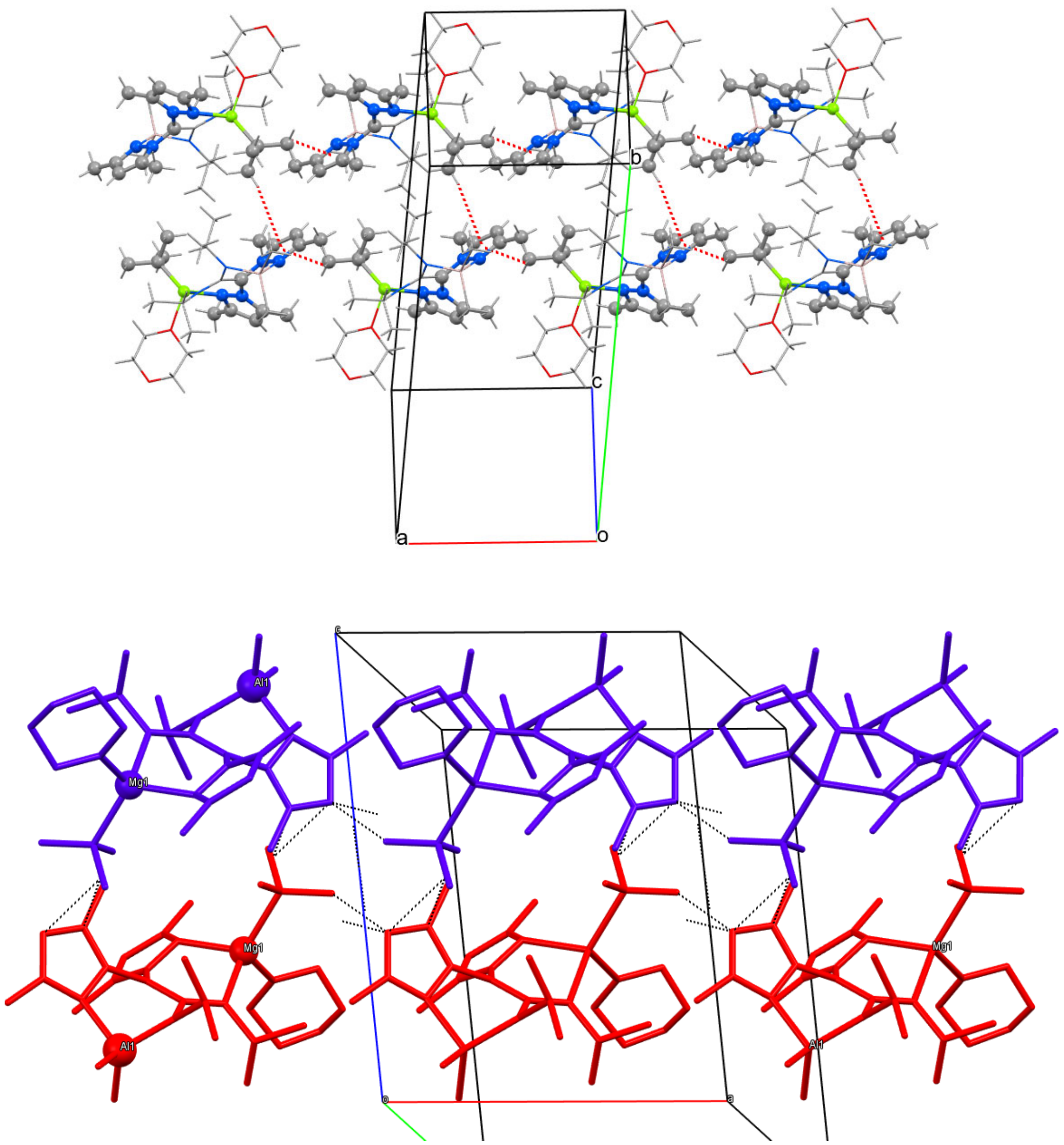

Figure S10. Views of *P* and *M* enantiomers and their alternating disposition along the c axis in the unit cell of [AlMe$_2$(pbpamd$^-$)Mg^{t}Bu{κ^1-O-(OC$_4$H$_8$O)}] (**2a**).

10. Experimental details for the ring-opening polymerization of poly(lactide)s.

General procedures for catalytic experiments

Polymerizations of lactides (L- or *rac*-LA) were performed on a Schlenk line in a flame-dried Schlenk tube equipped with a magnetic stirrer. The Schlenk tubes were charged in a glovebox with the required amount of lactide and then attached to the vacuum line. The required amount of initiator was weighed in a 5 mL vial, tapped, taken out of the glovebox and left near the Schlenk tube. The lactide was dissolved in the appropriate amount of solvent and temperature equilibration was ensured in the Schlenk tube (when lower than 23 ºC) by stirring the solution for 25 min in a bath. Next, the appropriate amount of initiator was directly added to the Schlenk tube and polymerization times were measured from that point. Polymerizations were stopped by injecting a solution of acetic acid in water (0.35 M). Polymers were precipitated in methanol, filtered off, redissolved en dichloromethane and reprecipitated in methanol, and dried in vacuo to a constant weight.

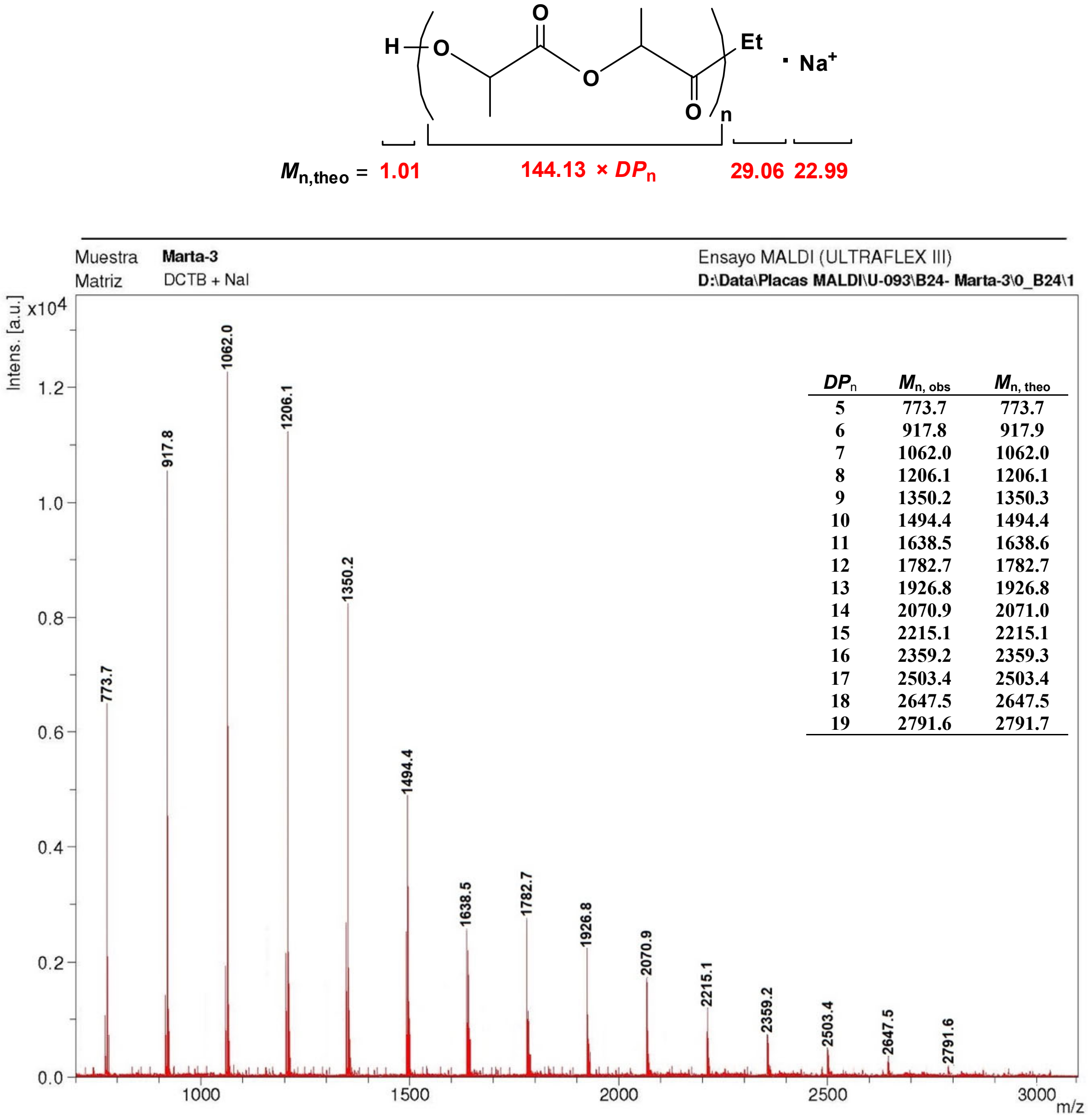

DP_n	$M_{n,\,obs}$	$M_{n,\,theo}$
5	773.7	773.7
6	917.8	917.9
7	1062.0	1062.0
8	1206.1	1206.1
9	1350.2	1350.3
10	1494.4	1494.4
11	1638.5	1638.6
12	1782.7	1782.7
13	1926.8	1926.8
14	2070.9	2071.0
15	2215.1	2215.1
16	2359.2	2359.3
17	2503.4	2503.4
18	2647.5	2647.5
19	2791.6	2791.7

Figure S11. Selected area of the MALDI-ToF mass spectrum of a PLA sample obtained on using [$AlMe_2$(pbpamd$^-$)MgEt{κ^1-O-(OC_4H_8O)}] (**1a**) with [**1a**]$_0$:[*rac*-LA]$_0$ = 30, 63% conversion, after quenching wet $CDCl_3$. Theoretical molecular weights calculated according to the equation: $M_n = (DP_n \times M_{wLA}) + M_{wHEt} + M_{wNa}$, where DP_n is the degree of polymerization, M_{wLA} = 144.13 g·mol^{-1}, M_{wHEt} = 30.07 g·mol^{-1} and M_{wNa} = 22.99 g·mol^{-1}.

The distribution in the spectrum indicates the existence of a single family of polymer chains capped by –CH(CH_3)OH and O–CH(CH_3)–C(O)–Et *termini*, corresponding to oligomers of formula H(OCHMeCO)$_{2n}$(–CH_2–CH_3)·Na^+ (n = 5 to 19) with consecutive peaks separated by increments of 144.14 Da. Moreover, neither intermolecular ester-exchange (transesterification) reactions nor cyclic oligomers were detected.

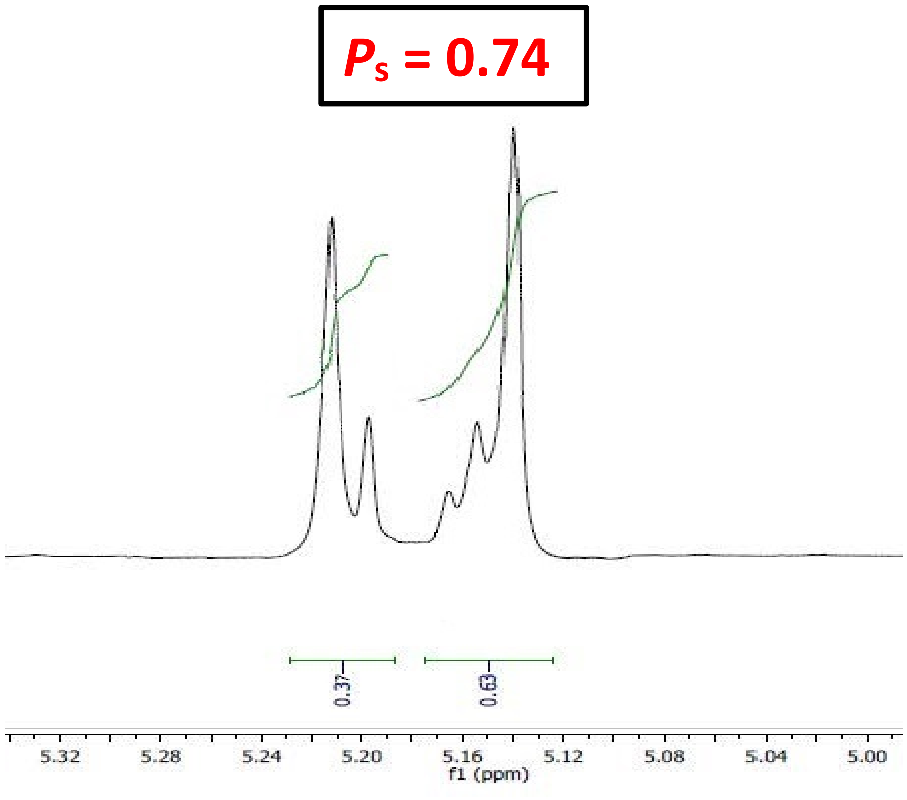

(a)

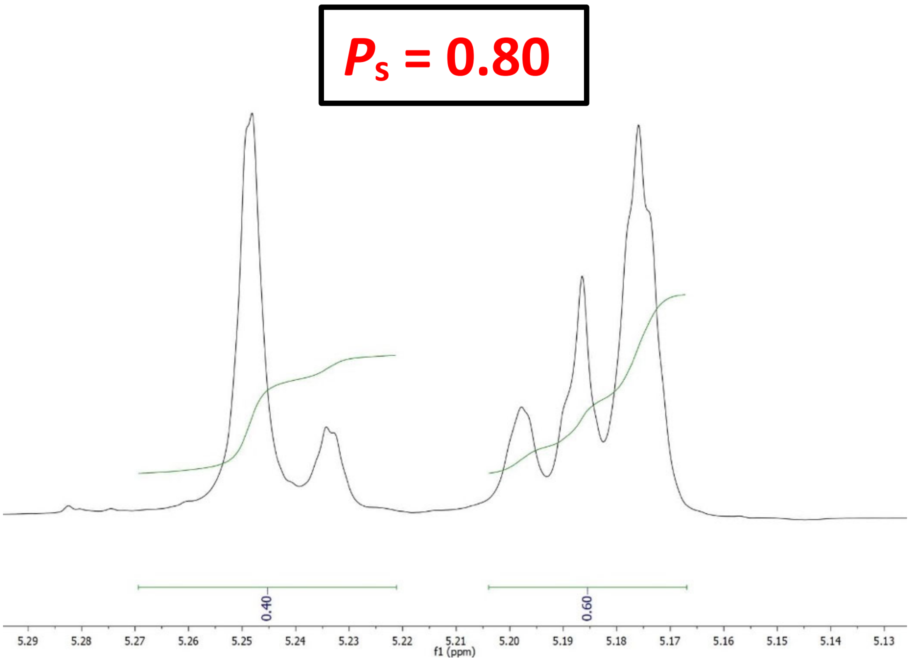

(b)

Figures S12. Homodecoupled ^{1}H NMR spectrum of poly(*rac*-lactide)s prepared by [$AlMe_2$(pbpamd$^-$)MgEt{κ^1-O-(OC_4H_8O)}] (**1a**) at (*a*) at 23 ºC and (*b*) at 0 ºC (Table 1, entries 4-5 and 7, respectively).

11. Kinetic investigations for the ring-opening polymerization of poly(L-lactide)s

Typical kinetic experiment procedure

The appropriate mass of catalyst **1a** was added as solid to a 0.4 M toluene solution of L-LA under a N_2 atmosphere. The initial monomer/catalyst ratio was $[\text{L-LA}]_0/[\mathbf{1a}]_0 = 400$, and the initial catalyst $[\mathbf{1a}]_0 = 1.0$ mM was modified up to 2.5 mM. At appropriate time intervals, 0.5 mL aliquots were removed using a syringe and quickly quenched into 5 mL vials with wet $CDCl_3$ (6 drops). The aliquots were then dried to constant weight in vacuo and analyzed by ^{1}H NMR spectroscopy. The standard error associated with the kinetic parameters was calculated by the standard deviation in slope and intercept for each regression analysis.

SUPPORTING INFORMATION TO
Kinetics analysis

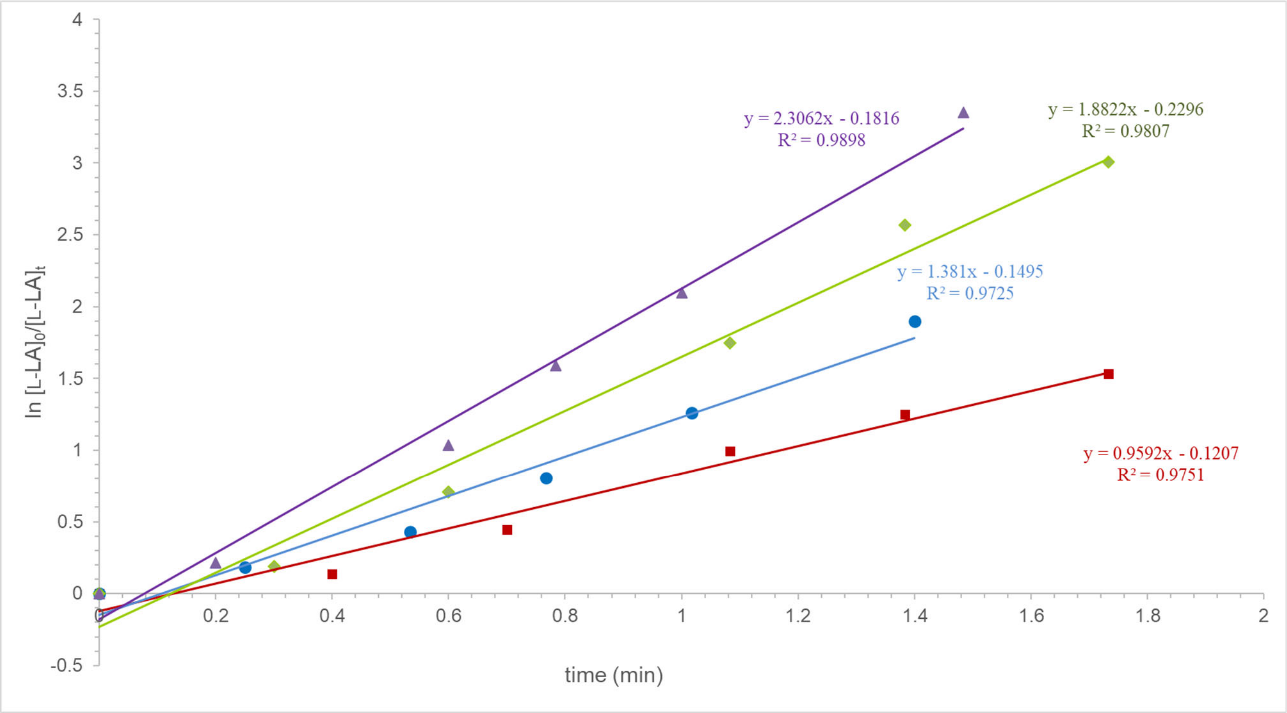

Figure S13. Pseudo-first-order kinetic plots for the polymerizations in toluene at room temperature employing [AlMe$_2$(pbpamd$^-$)MgEt{κ^1-O-(OC$_4$H$_8$O)}] (**1a**) as catalysts, with L-LA at [LA]$_0$ = 0.4 M.

In all cases, the linearity of the semi-logarithmic plots of ln ([L-LA]$_0$/[L-LA]$_t$) versus reaction time for catalyst **1a** at room temperature, employing different initial catalyst concentrations, shows that the propagations were first order with respect to L-LA monomer (Figure S13) (square correlation coefficients ≥ 0.97).

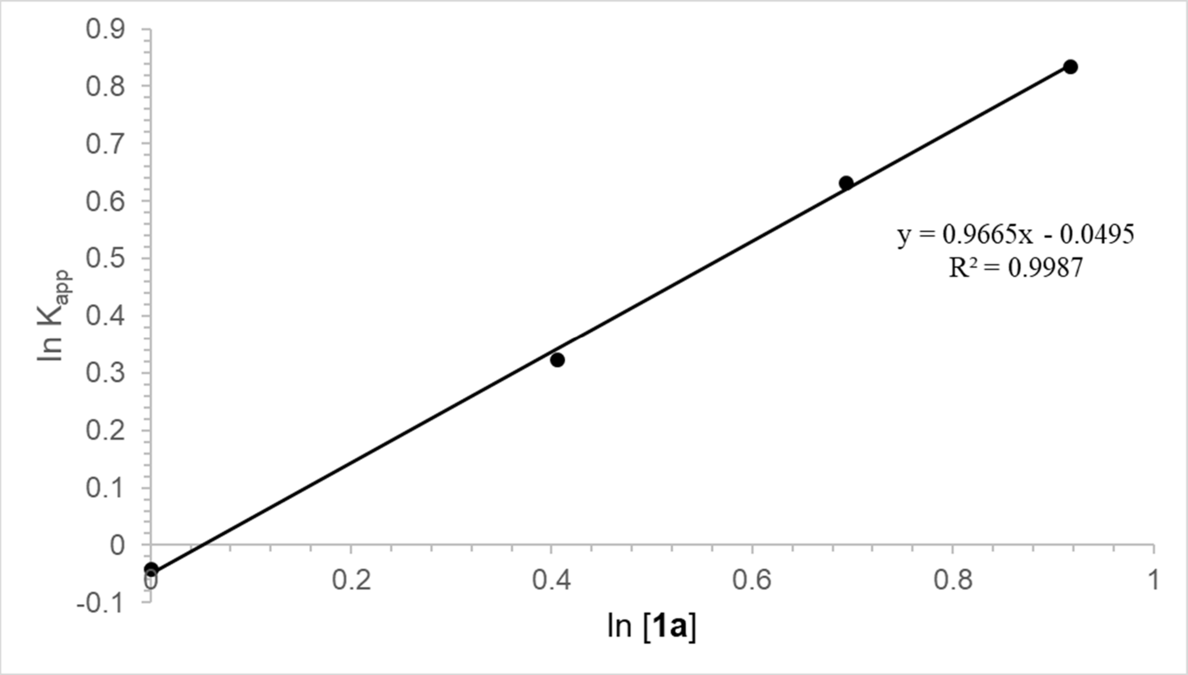

Figure S14. Plots of ln k_{app} versus ln $[\mathbf{1a}]_0$ for the polymerization of L-LA employing initiator $[AlMe_2(pbpamd^-)MgEt\{\kappa^1\text{-}O\text{-}(OC_4H_8O)\}]$ (**1a**) in toluene at room temperature, with $[\text{L-LA}]_0$ = 0.4 mol/L.

The kinetic dependence on the catalyst concentration (n) and the propagation rate constant (k_p) confirms that the reaction is also first order in catalyst **1a** at 23 ºC (Figure S14). These values prove that the polymerization of L-LA mediated by these initiators obeys an overall second-order rate kinetic law of the form:

$$- d[\text{L-LA}]/dt = k_p[\mathbf{1a}]^1[\text{L-LA}]^1$$

Table S5. Rate constant dependence on the initial concentration of **1a** for the ROP of L-LA at room temperature in toluene.

$[1a]_0 \times 10^3$ (M)	$k_{app} \times 10^2$ (s^{-1})	k_p ($M^{-1} \cdot s^{-1}$)	n
1.0	1.6 ± 0.1	15.9 ± 0.2	0.97 ± 0.03
1.5	2.3 ± 0.2		
2.0	3.1 ± 0.2		
2.5	3.8 ± 0.2		

12. References

[1] Alonso-Moreno, C.; Garcés, A.; Sánchez-Barba, L. F.; Fajardo, M.; Fernández-Baeza, J.; Otero, A.; Lara-Sánchez, A.; Antiñolo, A.; Broomfield, L.; López-Solera, M. I.; Rodríguez, A. M. *Organometallics* **2008**, 27, 1310-1321.

[2] Sandström, J. *Dynamic NMR spectroscopy*, Academic Press, London; New York, 1982.

[3] Gaussian 16, Revision B.01, Frisch, M. J.; Trucks, G. W.; Schlegel, H. B.; Scuseria, G. E.; Robb, M. A.; Cheeseman, J. R.; Scalmani, G.; Barone, V.; Petersson, G. A.; Nakatsuji, H.; Li, X.; Caricato, M.; Marenich, A. V.; Bloino, J.; Janesko, B. G.; Gomperts, R.; Mennucci, B.; Hratchian, H. P.; Ortiz, J. V.; Izmaylov, A. F.; Sonnenberg, J. L.; Williams-Young, D.; Ding, F.; Lipparini, F.; Egidi, F.; Goings, J.; Peng, B.; Petrone, A.; Henderson, T.; Ranasinghe, D.; Zakrzewski, V. G.; Gao, J.; Rega, N.; Zheng, G.; Liang, W.; Hada, M.; Ehara, M.; Toyota, K.; Fukuda, R.; Hasegawa, J.; Ishida, M.; Nakajima, T.; Honda, Y.; Kitao, O.; Nakai, H.; Vreven, T.; Throssell, K.; Montgomery, J. A., Jr.; Peralta, J. E.; Ogliaro, F.; Bearpark, M. J.; Heyd, J. J.; Brothers, E. N.; Kudin, K. N.; Staroverov, V. N.; Keith, T. A.; Kobayashi, R.; Normand, J.; Raghavachari, K.; Rendell, A. P.; Burant, J. C.; Iyengar, S. S.; Tomasi, J.; Cossi, M.; Millam, J. M.; Klene, M.; Adamo, C.; Cammi, R.; Ochterski, J. W.; Martin, R. L.; Morokuma, K.; Farkas, O.; Foresman, J. B.; Fox, D. J. Gaussian, Inc., Wallingford CT, 2016.

[4] a) A. D. Becke, *Phys. Rev. A* **1988**, *38*, 3098; b) J. P. Perdew, *Phys. Rev. B* **1986**, *33*, 8822.

[5] S. Grimme, J. Antony, S. Ehrlich, H. Krieg, *J. Chem. Phys.* **2010**, *132*, 154104.

[6] K. Eichkorn, O. Treutler, H. Öhm, M. Häser, R. Ahlrichs, *Chem. Phys. Lett.* **1995**, *242*, 652.

[7] a) S. Miertuš, E. Scrocco and J. Tomasi, *Chem. Phys.* **1981**, *55*, 117; b) J. L. Pascual-Ahuir, E. Silla, I. Tuñón, *J. Comput. Chem.* **1994**, *15*, 1127; c) V. Barone, M. Cossi, *J. Phys. Chem. A* **1998**, *102*, 1995.

[8] K. Wolinski, J. F. Hilton, P. Pulay, *J. Am. Chem. Soc.* **1990**, *112*, 8251

[9] SAINT v8.37, Bruker-AXS (2016), APEX3 v2016.1.0. Madison, Wisconsin, USA.

[10] SADABS, Krause, L., Herbst-Irmer, R., Sheldrick, G. M. & Stalke, D. *J. Appl. Crystallogr.* **2015**, *48*, 3.

[11] a) L. J. Farrugia, *J. Appl. Cryst.*, **2012**, *45*, 849. b) Dolomanov, O.V., Bourhis, L.J., Gildea, R.J, Howard, J.A.K. & Puschmann, H. c). G. M. Sheldrick, SHELX-2014, Program for Crystal Structure Refinement, University of Göttingen, Göttingen, Germany, 2014.
